# Predicting Health Material Accessibility: Development of Machine Learning Algorithms

**DOI:** 10.2196/29175

**Published:** 2021-09-01

**Authors:** Meng Ji, Yanmeng Liu, Tianyong Hao

**Affiliations:** 1 School of Languages and Cultures The University of Sydney Sydney Australia; 2 School of Computer Science South China Normal University Guangdong China

**Keywords:** health education materials, Chinese language, cognitive accessibility, readability, semantic features, health education, machine learning, prediction, accessibility, health text, cognition, accessibility, semantic, algorithm, health information

## Abstract

**Background:**

Current health information understandability research uses medical readability formulas to assess the cognitive difficulty of health education resources. This is based on an implicit assumption that medical domain knowledge represented by uncommon words or jargon form the sole barriers to health information access among the public. Our study challenged this by showing that, for readers from non-English speaking backgrounds with higher education attainment, semantic features of English health texts that underpin the knowledge structure of English health texts, rather than medical jargon, can explain the cognitive accessibility of health materials among readers with better understanding of English health terms yet limited exposure to English-based health education environments and traditions.

**Objective:**

Our study explores multidimensional semantic features for developing machine learning algorithms to predict the perceived level of cognitive accessibility of English health materials on health risks and diseases for young adults enrolled in Australian tertiary institutes. We compared algorithms to evaluate the cognitive accessibility of health information for nonnative English speakers with advanced education levels yet limited exposure to English health education environments.

**Methods:**

We used 113 semantic features to measure the content complexity and accessibility of original English resources. Using 1000 English health texts collected from Australian and international health organization websites rated by overseas tertiary students, we compared machine learning (decision tree, support vector machine [SVM], ensemble tree, and logistic regression) after hyperparameter optimization (grid search for the best hyperparameter combination of minimal classification errors). We applied 5-fold cross-validation on the whole data set for the model training and testing, and calculated the area under the operating characteristic curve (AUC), sensitivity, specificity, and accuracy as the measurement of the model performance.

**Results:**

We developed and compared 4 machine learning algorithms using multidimensional semantic features as predictors. The results showed that ensemble classifier (LogitBoost) outperformed in terms of AUC (0.858), sensitivity (0.787), specificity (0.813), and accuracy (0.802). Support vector machine (AUC 0.848, sensitivity 0.783, specificity 0.791, and accuracy 0.786) and decision tree (AUC 0.754, sensitivity 0.7174, specificity 0.7424, and accuracy 0.732) followed. Ensemble classifier (LogitBoost), support vector machine, and decision tree achieved statistically significant improvement over logistic regression in AUC, sensitivity, specificity, and accuracy. Support vector machine reached statistically significant improvement over decision tree in AUC and accuracy. As the best performing algorithm, ensemble classifier (LogitBoost) reached statistically significant improvement over decision tree in AUC, sensitivity, specificity, and accuracy.

**Conclusions:**

Our study shows that cognitive accessibility of English health texts is not limited to word length and sentence length as had been conventionally measured by medical readability formulas. We compared machine learning algorithms based on semantic features to explore the cognitive accessibility of health information for nonnative English speakers. The results showed the new models reached statistically increased AUC, sensitivity, and accuracy to predict health resource accessibility for the target readership. Our study illustrated that semantic features such as cognitive ability–related semantic features, communicative actions and processes, power relationships in health care settings, and lexical familiarity and diversity of health texts are large contributors to the comprehension of health information; for readers such as international students, semantic features of health texts outweigh syntax and domain knowledge.

## Introduction

### Readability Matters

Health education materials provide important educational interventions to help increase the awareness of health risks. The recent outbreaks of the COVID-19 pandemic highlight the need to develop accessible health information, as health information appraisal has emerged as an issue in high-income countries [1]. The efficiency of health education materials largely depends on the readability and cognitive accessibility of the materials [2]. As such, the World Health Organization recommends several principles for developing health education materials regarding readability [3]. It is suggested that the readability level of medical information be lower than sixth grade for the public, and there should be easier material design for people with poor understanding capabilities [4-7]. However, studies indicate that many health education materials are more difficult than expected, leaving the layman readers encountering difficulties to comprehend the materials, which will inevitably compromise the efficiency of the health risk intervention [8-11].

Enhanced readability will improve the accessibility of health educational resources. Widely used readability assessment tools are medical readability formulas [12]. medical readability formulas measure health information readability based on word length or sentence length, assuming that the longer words and sentences are, the more difficult the health content is. These formulas are challenged by scholars due to its oversimplified factors considered in the calculations and inconsistency assessment results [13,14]. For health education texts, the cognitive difficulty in understanding medical information is caused not only by medical jargon and complex sentences but also by semantic meanings, which cannot be directly represented by word and sentence length alone [15-17]. However, readability estimation tools considering semantic features are few and underexplored. Readability estimation tools considering semantic features are in urgent need, especially for readers with better understanding of health terms yet limited exposure to English health education materials. These types of readers, represented by nonnative English speakers living in English-speaking countries, like the United States, Australia, New Zealand, or Canada, make up a large quantity of the population whose health education is of concern for the society [18-21]. These readers pose new challenges for medical readability assessment, as they normally have sufficient understanding of health terms yet limited exposure to English health education materials. In these cases, semantic features of English health texts rather than medical jargon would be suitable to estimate the cognitive accessibility of health materials.

Our study will address the challenges of using existing medical readability formulas to provide valid effective assessment of health information for readers with bilingual proficiency yet limited exposure to English health education traditions. We will introduce semantic features as indicators in cognitive accessibility evaluation. Compared with previous approaches that focus on morphological and syntactic features, we will explore the validity and effectiveness of using multidimensional semantic features (especially lexis related to English health education cultures) to analyze, model, and predict the cognitive accessibility of English health education materials. Improving cognitive accessibility of health education materials will provide a cost-effective approach to public health education. Improvement in cognitive accessibility of health education materials will contribute to social and health quality among readers from nonnative English speaking backgrounds [22].

### Data Sets and Feature Extraction

#### Material Collection and Classification

This paper collected health education materials in English from government, health agencies, and not-for-profit organizations in Australia, considering Australia is a typical migrant country with a large amount of nonnative English speakers living in the country. The source of the health education materials includes Department of Health in state governments like Western Australia, New South Wales, and Victoria, and not-for-profit organizations [23-26]. The topic of the materials is about infectious diseases like COVID-19, Ebola, plague, or Zika, as infectious disease education is urgent in need with the background of pandemic outbreaks in recent years. In total, 1000 health education articles were collected with a size of over 500,000 words. The types of materials are patient guidelines, fact sheets, and health topics, which are health education resources accessible by the public to improve their health awareness or health knowledge. For classification, we invited 4 international students studying in Australian universities as labelers to rate the readability of the collected materials. The labelers were aged between 25 and 30 years, nonnative English speakers with advanced English skills (International English Language Testing System test score 6.5 or greater), and they were born and grew up in non-English speaking countries with limited exposure to English health education materials. They were asked to classify the collected health texts independently into easy versus hard to understand categories, and the interrater agreement was high (Cohen kappa 0.705). The final classification contained two sets of texts: easy (n=495) versus difficult (n=505; original annotated data sets in Multimedia Appendix 1).

#### Material Annotation and Semantic Feature Extraction

The UCREL (University Centre for Computer Corpus Research on Language) Semantic Analysis System (USAS) was adopted to annotate health education materials and extract semantic features [27]. The system relies on several disambiguation methods including part-of-speech tagging, general likelihood ranking, multiword expression extraction, domain of discourse identification, and contextual rules, providing high annotation accuracy of English texts. USAS categorizes English words into 21 semantic groups, including general and abstract terms (group A); physical condition and bodily processes (group B); emotions (group D); food and drinks (group F); governmental activities (group G); residence, buildings, and habitats (group H); work and employment (group I); entertainment, sports, and activities (group K); life and living things (group L); movement, location, and transport (group M); numbers and measurements (group N); substances, materials, objects, and equipment (group O); education (group P); linguistic actions, states, and processes (group Q); social states, actions, and processes (group S); time (group T); geographical terms (group W); psychological actions, states, and processes (group X); science and technology (group Y); and names and grammatical words (group Z). With USAS, we collected 113 semantic features. In this study, we extracted these semantic features automatically from specialized English health materials to provide additional text information for developing machine learning algorithms.

### Statistical Analysis of Multidimensional Semantic Features in English Educational Health Texts

Multimedia Appendix 1 shows the results of a logistic regression of the entire annotated database. A total of 26 of the 113 semantic features were identified as statistically significant features contributing to the binary classification of health texts in terms of their understandability to the target readerships, international students in tertiary education. Several semantic features contributing to the higher understandability of health texts were identified. First, *informational coherence* through pronouns (Z8) is a large contributor to the cognitive accessibility of English health texts among non-English readers, even those with advanced English language skills. The *P* and the effect size of the semantic feature Z8 were <.001 and .91, respectively, suggesting a very significant difference between easy and difficult health texts in terms of the use of pronouns. The mean score of Z8 in health texts of higher understandability was 52.84, this dropped to 20.48 in health texts of low understandability. Further, in the logistic regression analysis, the odds ratio of Z8 (ratio of odds between difficult and easy texts, with easy text as the reference text class) was 0.928 (95% CI 0.905-0.951), indicating, with the increase of 1 standard unit of Z8, the odds of the health text being a difficult health reading over the odds of the text being an easy reading was 0.928. In terms of percentage change, the odds of the health text being a difficult text was 0.031 lower than the odds of the text being an easy reading for the target readers. Semantic features related to the *logical structure* (Z7 conditional expressions such as if) were identified as statistically significant (*P*=.01). The odds ratio of Z7 was 0.86 (95% CI 0.767-0.964), indicating that holding other textual features unchanged, with the increase of one word in the Z7 class, the odds of the health text being a difficult text was 86% over the odds of the text being an easy reading.

The logistic regression result (Multimedia Appendix 1) also identified 12 semantic features as statistically significant contributors to the perceived difficulty of English health texts. Typical examples were B3 (medicines and medical treatment; odds ratio Exp(B) 1.041, 95% CI 1.012-1.071; *P*=.005), Z99 (out-of-dictionary words; odds ratio Exp(B) 1.011, 95% CI 1.004-1.018; *P*=.001), L2 (living creatures: animals, microorganism, virus, bacteria, etc; odds ratio Exp(B) 1.080, 95% CI 1.005-1.162; *P*=.036), and W5 (environmental terms: pollutants, carcinogens, inhalable particles, etc.; odds ratio Exp(B) 2.441, 95% CI 1.173-5.077; *P*=.017). These semantic features measured *lexical familiarity and diversity* of English health texts, which is another important dimension of the assessment of medical and health lexis understandability. For example, the relatively large odds ratios (2.441, 95% CI 1.173-5.077) of W5 encompassing terms related to environmental exposure and health risks indicates that, with the increase of one word in this particular category, the odds of a health text being a difficult text over the odds of the text being an easy text for the target readers was 2.441, or in terms of percentage change, this represents an increase of 144.1% of the text from an easy text to a very difficult health reading. To a lesser extent, the odds ratio of 1.080 of L2 (living creatures including microorganisms) indicates that with the increase of one word in this class, the perceived difficulty level (hard-to-understand class) of the health text increased by a mean 8.0% (95% CI 0.5%-16.2%) depending on the vocabulary range of English health terms of the readers. Semantic features relating more *abstract concepts and higher cognitive abilities* were detected as statistically significant contributors to the perceived difficulty of health texts. These include A11 (abstract terms denoting importance, significance, noticeability, or markedness; odds ratio 1.219, 95% CI 1.070-1.388; *P*=.003). This means that with the increase of one unit in the A11 class, the odds of the health text being seen as a hard-to-understand text over the text being seen as an easy text was 1.219, or an increase of 21.9%.

In the next section, we will use these predictor variables to compare the performance of machine learning algorithms in analyzing and predicting the cognitive accessibility of English health materials for the intended readership of international tertiary students.

## Methods

Using machine learning algorithms and natural language processing tools to analyze and predict the understandability levels of health information has been gaining momentum. Zheng and Yu [28] used surface text features and word embeddings to support vector machine (SVM) algorithms to assess and rank the readability levels of electronic health records and Wikipedia articles. Venturi et al [29] also applied SVM to evaluate and predict the cognitive difficulty of medical informed consent forms in Italian. They used natural language features such as part of speech, type token ratio, noun verb ratio, average parse tree depth, main versus subordinate clauses distribution, distribution of verbal roots with explicit subject, and other syntactic and grammatical features related to Italian linguistic complexity. However, few existing studies have explored the effects of semantic features on the understandability of health information as our study did.

**Figure 1:**
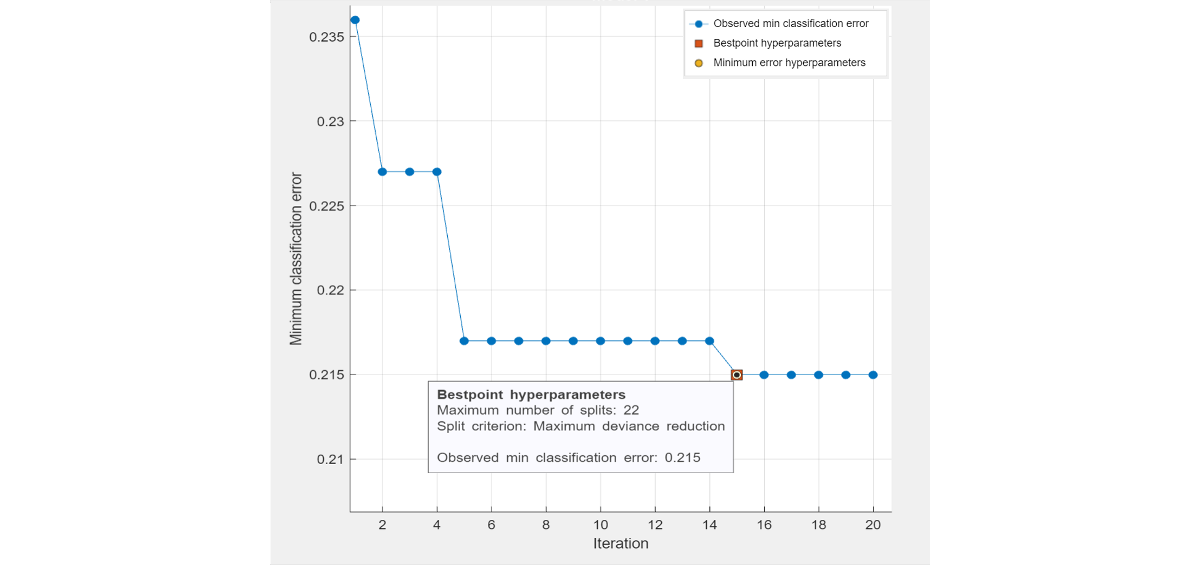
Hyperparameter tuning (decision tree).

**Figure 2:**
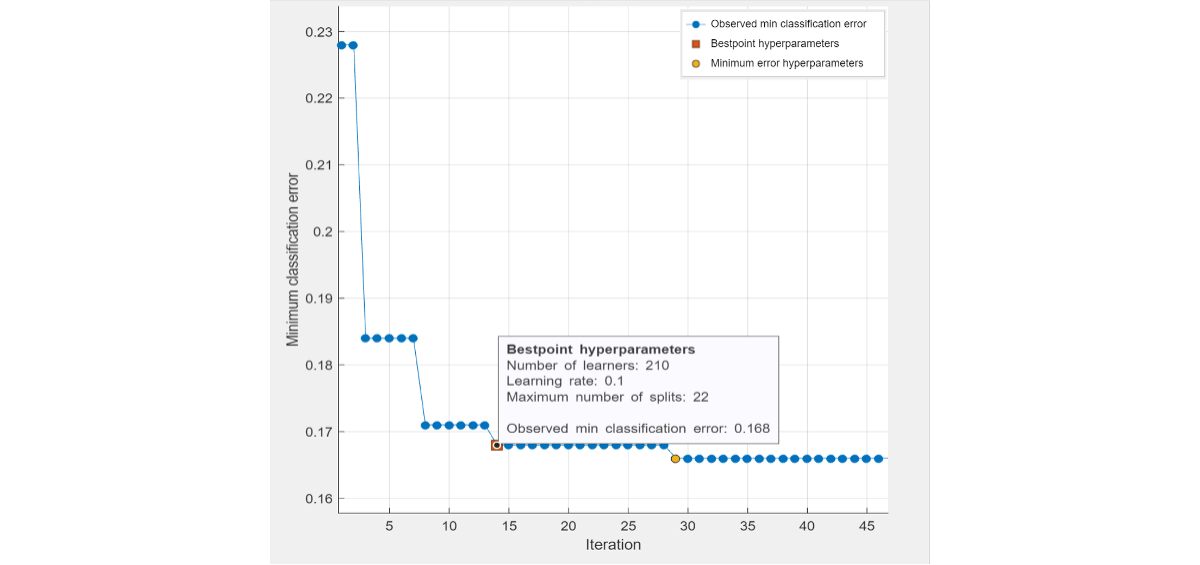
Hyperparameter tuning (ensemble classifier).

**Figure 3:**
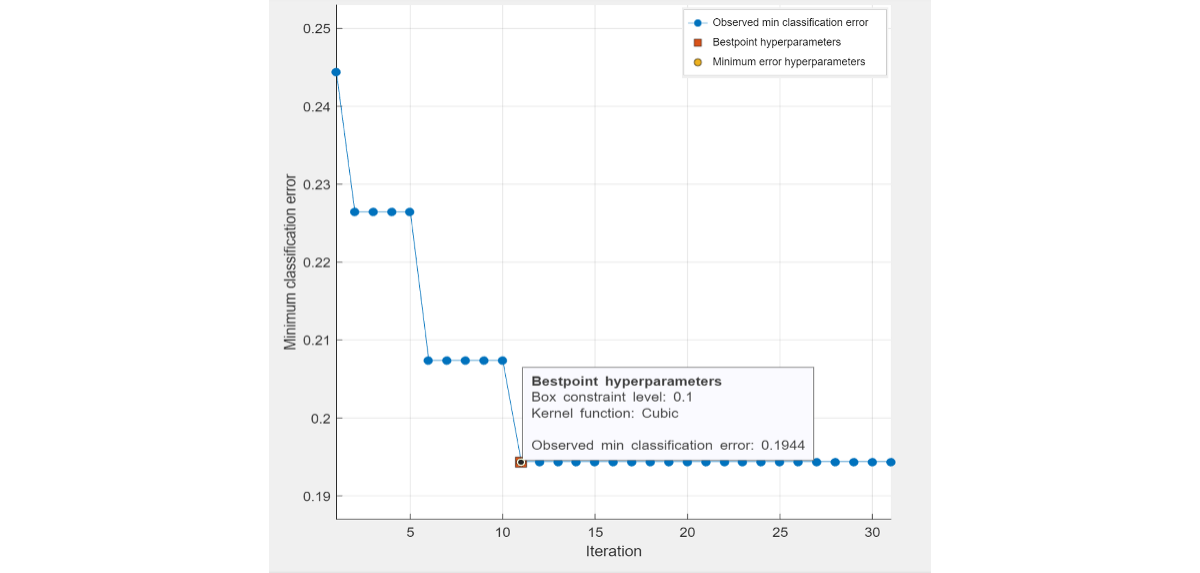
Hyperparameter tuning (support vector machine).

The four machine learning methods used in this study were ensemble classifier, SVM, decision tree classifier, and logistic regression classifier. Ensemble classifier (LogitBoost), SVM, and decision tree are optimizable models, as their hyperparameters can be fine-tuned through automatic grid searches to achieve minimal classification errors. For a decision tree classifier, the best-point hyperparameters (Figure 1) were the maximum number of tree splits (n=22) based on maximum deviance reduction. The observed minimal classification error of the optimized decision tree model was 0.215. For an ensemble classifier, the best-point hyperparameters (Figure 2) reached an observed minimum classification error of 0.168. The optimized hyperparameters were the ensemble method (LogitBoost), number of learners (n=210), learning rate (0.1), and maximum number of splits (n=22). For SVM, the best-point hyperparameters (Figure 3) were box constraint level (0.1), kernel function (cubic). The observed minimum classification error was 0.1944, lower than the optimized decision tree model (with a difference of 0.0206) but higher than the optimized ensemble classifier (with a difference of 0.0264).

## Results

The predictive performance of the four machine learning algorithms using multidimensional semantic features as predictor variables is shown in Table 1, and the results of the pairwise corrected resampled *t* test are shown in Tables 2-5. The mean scores and standard deviations of the area under the operating characteristic curve (AUC), sensitivity, specificity, and accuracy were obtained through 5-fold cross-validation. The cross-validation divided the entire data set into 5 folds of equal size. In each iteration, 4 folds were used for the training data, and the remaining fold was used as the testing data. As a result, on completion of the 5-fold cross-validation, each fold was used as the testing data exactly once. We used paired-sample comparisons to investigate the area under the operating characteristic curve (AUC), sensitivity, specificity, and accuracy differences of four machine learning algorithms (n=6; α=.05).

**Table 1:** Performance of the machine learning models using multidimensional semantic features as predictors.

Algorithm	AUC^a^, mean (SD)	Sensitivity, mean (SD)	Specificity, mean (SD)	Accuracy, mean (SD)
LR^b^	0.614 (0.0554)	0.6282 (0.0597)	0.5724 (0.0733)	0.6010 (0.0523)
SVM^c^	0.848 (0.0172)	0.7830 (0.0368)	0.7910 (0.0420)	0.7860 (0.0153)
DT^d^	0.754 (0.0377)	0.7174 (0.0719)	0.7424 (0.0589)	0.732 (0.0317)
ENS^e^	0.858 (0.041)	0.787 (0.057)	0.813 (0.046)	0.802 (0.032)

^a^AUC: area under the operating characteristic curve.

^b^LR: logistic regression.

^c^SVM: support vector machine.

^d^DT: decision tree.

^e^ENS: ensemble classifier (LogitBoost).

**Table 2:** Pairwise corrected resampled *t* test of area under the curve differences (using multidimensional semantic features as predictor variables).

Pairs	Mean difference (SD)	Standard error mean	95% CI	*t* test (*df*)	*P* value
LR^a^ vs SVM^b^	–0.2340 (0.0669)	0.0299	–0.3171 to –0.1509	–7.817 (4)	.001
LR vs DT^c^	–0.1460 (0.0551)	0.0246	–0.2144 to –0.0777	–5.931 (4)	.004
LR vs ENS^d^	–0.2440 (0.0564)	0.0252	–0.3140 to –0.1740	–9.675 (4)	.001
SVM vs DT	0.0880 (0.0192)	0.0086	–0.0641 to 0.1119	10.230 (4)	.001
SVM vs ENS	–0.0100 (0.0374)	0.0167	–0.0565 to –0.0365	–0.598 (4)	.582
DT vs ENS	–0.0980 (0.0192)	0.0086	–0.1219 to –0.0741	–11.392 (4)	<.001

^a^LR: logistic regression.

^b^SVM: support vector machine.

^c^DT: decision tree.

^d^ENS: ensemble classifier (LogitBoost).

**Table 3:** Pairwise corrected resampled *t* test of sensitivity differences (using multidimensional semantic features as predictor variables).

Pairs	Mean difference (SD)	Standard error mean	95% CI	*t* test (*df*)	*P* value
LR^a^ vs SVM^b^	–0.1548 (0.0303)	0.0135	–0.1924 to –0.1172	–11.429 (4)	<.001
LR vs DT^c^	–0.1002 (0.0720)	0.0322	–0.1896 to –0.0108	–3.111 (4)	.036
LR vs ENS^d^	–0.1588 (0.0945)	0.0423	–0.2761 to –0.0414	–3.756 (4)	.020
SVM vs DT	0.0546 (0.0697)	0.0312	–0.0319 to 0.1411	1.752 (4)	.155
SVM vs ENS	–0.0040 (0.0855)	0.0382	–0.1102 to –0.1022	–0.105 (4)	.922
DT vs ENS	–0.0586 (0.0371)	0.0166	–0.1046 to –0.0126	–3.535 (4)	.024

^a^LR: logistic regression.

^b^SVM: support vector machine.

^c^DT: decision tree.

^d^ENS: ensemble classifier (LogitBoost).

**Table 4:** Pairwise corrected resampled *t* test of specificity differences (using multidimensional semantic features as predictor variables).

Pairs	Mean difference (SD)	Standard error mean	95% CI	*t* test (*df*)	*P* value
LR^a^ vs SVM^b^	–0.2186 (0.0968)	0.0433	–0.3389 to –0.0984	–5.047 (4)	.007
LR vs DT^c^	–0.1720 (0.0822)	0.0368	–0.2741 to –0.0699	–4.679 (4)	.009
LR vs ENS^d^	–0.2410 (0.0677)	0.0303	–0.3251 to –0.1569	–7.959 (4)	.001
SVM vs DT	0.0466 (0.1059)	0.0474	–0.0849 to 0.1781	0.984 (4)	.381
SVM vs ENS	–0.0224 (0.0918)	0.0411	–0.1364 to –0.0916	–0.545 (4)	.614
DT vs ENS	–0.0690 (0.0334)	0.0149	–0.1105 to –0.0275	–4.619 (4)	.010

^a^LR: logistic regression.

^b^SVM: support vector machine.

^c^DT: decision tree.

^d^ENS: ensemble classifier (LogitBoost).

**Table 5:** Pairwise corrected resampled *t* test of accuracy differences (using multidimensional semantic features as predictor variables).

Pairs	Mean difference (SD)	Standard error mean	95% CI	*t* test (*df*)	*P* value
LR^a^ vs SVM^b^	–0.1850 (0.0507)	0.0227	–0.2480 to –0.1220	–8.152 (4)	.001
LR vs DT^c^	–0.1370 (0.0482)	0.0215	–0.1968 to –0.0771	–6.360 (4)	.003
LR vs ENS^d^	–0.2010 (0.0549)	0.0246	–0.2692 to –0.1328	–8.182 (4)	.001
SVM vs DT	0.0480 (0.0295)	0.0132	0.0114 to 0.0846	3.639 (4)	.022
SVM vs ENS	–0.0160 (0.0366)	0.0164	–0.0615 to 0.0295	–0.976 (4)	.384
DT vs ENS	–0.0640 (0.0148)	0.0066	–0.0823 to –0.0457	–9.704 (4)	.001

^a^LR: logistic regression.

^b^SVM: support vector machine.

^c^DT: decision tree.

^d^ENS: ensemble classifier (LogitBoost).

**Figure 4:**
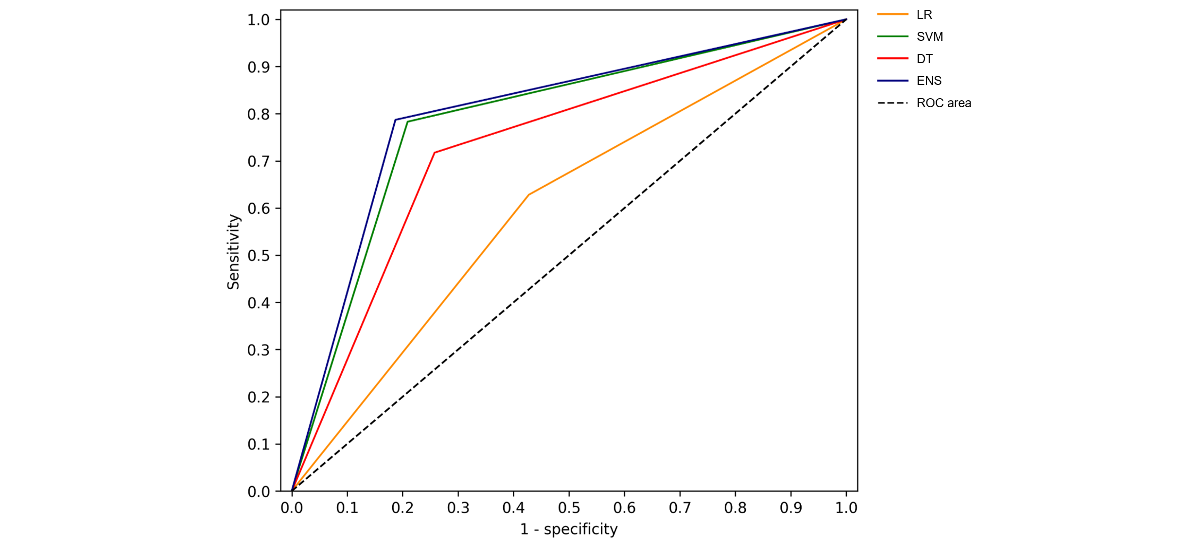
Mean ROC curve for machine learning algorithms. DT: decision tree; LR: logistic regression; ROC: receiver operating characteristic; SVM: support vector machine.

Table 2 shows that, in terms of AUC, ensemble classifier (LogitBoost), decision tree, and SVM reached statistically improved AUC over logistic regression (0.614): ensemble classifier (0.858; *P*=.001), decision tree (0.754; *P*=.004), and SVM (0.848, *P*=.001). In terms of sensitivity (Table 3), ensemble classifier (0.787, *P*=.020), decision tree (0.7174, *P*=.036), and SVM (0.783; *P*<.001) reached statistically significant improvement over logistic regression (0.6282). In terms of model specificity (Table 4), ensemble classifier, decision tree, and SVM all reached statistically improved specificity over logistic regression (0.5724): ensemble classifier (0.813; *P*=.001), decision tree (0.7424; *P*=.009), and SVM (0.791; *P*=.007). Lastly, with regard to model overall accuracy (Table 5), again, LogitBoost, decision tree, and SVM outperformed logistic regression (0.601): ensemble classifier (0.802; *P*=.001), decision tree (0.732; *P*=.003), and SVM (0.786; *P*=.001). Comparing SVM, ensemble classifier and decision tree, the former two algorithms outperformed decision tree consistently in AUC (*P*=.001 and *P*<.001, respectively), and accuracy (*P*=.022 and *P*=.001, respectively). Only ensemble classifier outperformed decision tree significantly in terms of model sensitivity (*P*=.024), and specificity (*P*=.010), using the paired-sample comparisons (n=6; α=.05). These results suggest that, when using semantic features as predictor variables, the most stable and highest-performing algorithm is ensemble classifier (LogitBoost), followed by SVM. Ensemble classifier, decision tree, and SVM all achieved statistically significant improvement over logistic regression in AUC, specificity, sensitivity, and accuracy. SVM did not improve significantly over decision tree in terms of sensitivity and specificity, but ensemble classifier did. Overall, the best AUC, sensitivity, specificity, and accuracy were achieved by LogitBoost as an ensemble classifier (Figure 4).

## Discussion

### Principal Findings

The understandability of health texts has long been assessed using medical readability formulas. This has simplified and limited the discussion of health information accessibility to two known barriers (ie, medical jargon and syntactic features). Existing research has been limited in exploring these issues despite methodological innovation in applying and leveraging machine learning algorithms and natural language processing tools in this field. Our study explored health information accessibility using semantic features of health information that are less studied. This was in line with clinical insights into patient-oriented health education, which identified multiple textual features as highly relevant to the understanding of specialized health information. However, few existing studies have attempted to translate recent clinical guidelines and insights to quantitative computational studies using linguistic features related to the semantic content as exemplified in our study. Using semantic annotation tools, we explored effects of various semantic features on the understandability of health texts for the target readers.

In the multiple machine learning algorithm comparison, the importance of semantic features was verified. It was found that, in the algorithm comparison experiments, using multidimensional semantic features as predictor variables, LogitBoost achieved the highest performance in terms of AUC, sensitivity, specificity, and accuracy, which were statistically significant large improvements (measured in pairwise resampled *t* tests). Among the 4 algorithms used, AUCs, sensitivity, specificity, and accuracy were consistently high when using multidimensional semantic features as predictors variables. This finding suggests that multidimensional semantic features are large contributors to the cognitive accessibility of English health texts among readers with English proficiency but limited exposure to English health education traditions (indicated by less familiarity of relevant health lexis and abstract concepts).

Considering that the readership under study were educated international tertiary students who had less barriers to understand and analyze complex English syntactic structures but had limited exposure to English-based health education environments, our study shows that, for readers from this background of health literacy and education level, informational coherence and logical structure were large contributors to the ease of health texts. Features of health-related lexical familiarity and diversity or those indicating abstract concepts or requiring higher cognitive abilities can significantly increase the difficulty of English health information for readers from non-English speaking and distinct health education backgrounds, despite their English proficiency from tertiary education.

In the development of effective reader-oriented health educational resources, enhancing semantic features, which were identified as large contributors to cognitive ease, can lead to more beneficial reading experiences among the target readers. Textual interventions can be effectively introduced to reduce the cognitive load of health texts, such as health lexical diversity (especially those of large odds ratios such as environmental exposure and health risks), or those requiring higher cognitive abilities, such as abstract terms denoting importance, significance, noticeability or markedness of health events and situations. These semantic features can significantly increase the difficulty and inaccessibility of English health education resources among international students, as these semantic features require greater, more sustained exposure to English public health education traditions.

### Limitations and Future Research

Our study was based on a small group of international students from native Chinese speaking backgrounds. Their rating of the cognitive understandability of English health texts could have been biased by their shared cultural backgrounds. This was, however, intended to control for cultural demographic diversity in our study. Whether this finding applies to other cohorts of international tertiary students remains to be evaluated through similar experiment design. Another considerable limitation of our study is the lack of explanation by the machine learning–based prediction. In future research, we aim to develop more explainable machine learning models to increase the interpretability of the prediction results.

### Conclusion

Our study showed that cognitive accessibility of English health texts is not limited to medical jargon and complex syntax such as long words and sentences conventionally measured by medical readability formulas. We compared machine learning algorithms using multiple semantic features to explore the cognitive accessibility of health information from multiple semantic perspectives. The results showed the strength of our models in terms of consistently high AUC, sensitivity, specificity, and accuracy to predict health resource accessibility for the target readers, indicating that semantics contribute to the comprehension of health information and that, for readers with advanced education, semantic features that underpin the English-based health education can outweigh syntax and specialized medical domain knowledge.

## Appendix

Multimedia Appendix 1 Variables in the logistic regression of health text understandability membership.

## Abbreviations

AUC: area under the operating characteristic curve
SVM: support vector machine
UCREL: University Centre for Computer Corpus Research on Language
USAS: UCREL Semantic Analysis System
