# Correction: Predicting Health Material Accessibility: Development of Machine Learning Algorithms

**DOI:** 10.2196/33385

**Published:** 2021-09-21

**Authors:** Meng Ji, Yanmeng Liu, Tianyong Hao

**Affiliations:** 1 School of Languages and Cultures The University of Sydney Sydney Australia; 2 School of Computer Science South China Normal University Guangdong China

In “Predicting Health Material Accessibility: Development of Machine Learning Algorithms” (JMIR Med Inform 2021;9(9):e29175) the authors noted some errors. The following changes have been made to correct these errors:

**Author Metadata**

In the originally published paper, Affiliation 1 appeared as follows:

School of Languages and Culture, The University of Sydney, Sydney, Australia

It is now corrected as follows:

School of Languages and Cultures, The University of Sydney, Sydney, Australia

**Abstract**

- Under "Methods," the phrase "*We applied 10-fold cross-validation on the whole data set...*" has been replaced by "*We applied 5-fold cross-validation on the whole data set....*"
- Under "Results," the sentences "*The results showed that ensemble tree (LogitBoost) outperformed in terms of AUC (0.97), sensitivity (0.966), specificity (0.972), and accuracy (0.969). Decision tree (AUC 0.924, sensitivity 0.912, specificity 0.9358, and accuracy 0.924) and SVM (AUC 0.8946, sensitivity 0.8952, specificity 0.894, and accuracy 0.8946) followed closely. Decision tree, ensemble tree, and SVM achieved statistically significant improvement over logistic regression in AUC, specificity, and accuracy. As the best performing algorithm, ensemble tree reached statistically significant improvement over SVM in AUC, specificity, and accuracy, and statistically significant improvement over decision tree in sensitivity*" have been replaced by "*The results showed that ensemble classifier (LogitBoost) outperformed in terms of AUC (0.858), sensitivity (0.787), specificity (0.813), and accuracy (0.802). Support vector machine (AUC 0.848, sensitivity 0.783, specificity 0.791, and accuracy 0.786) and decision tree (AUC 0.754, sensitivity 0.7174, specificity 0.7424, and accuracy 0.732) followed. Ensemble classifier (LogitBoost), support vector machine, and decision tree achieved statistically significant improvement over logistic regression in AUC, sensitivity, specificity, and accuracy. Support vector machine reached statistically significant improvement over decision tree in AUC and accuracy. As the best performing algorithm, ensemble classifier (LogitBoost) reached statistically significant improvement over decision tree in AUC, sensitivity, specificity, and accuracy.*"

**Introduction**

- Under "Material Collection and Classification," the last sentence "*The final classification contained two sets of texts: easy (n=499) versus difficult (n=501;...*" has been replaced by "*The final classification contained two sets of texts: easy (n=495) versus difficult (n=505;....*"
- Under "Material Annotation and Semantic Feature Extraction," the sentence "*With USAS, we collected 108 semantic features*" has been replaced by "*With USAS, we collected 113 semantic features.*"
- Under "Statistical Analysis of Multidimensional Semantic Features in English Educational Health Texts," in the first paragraph, the sentence "*A total of 29 of the 113 semantic features were identified as statistically significant…*" has been replaced by "*A total of 26 of the 113 semantic features were identified as statistically significant....*"
- Under "Statistical Analysis of Multidimensional Semantic Features in English Educational Health Texts," in the first paragraph, the sentence "*The mean score of Z8 in health texts of higher understandability was 52.91, this dropped to 20.15 ...*" has been replaced by "*The mean score of Z8 in health texts of higher understandability was 52.84, this dropped to 20.48....*"
- Under "Statistical Analysis of Multidimensional Semantic Features in English Educational Health Texts," in the first paragraph, the sentence "*…was 0.929 (95% CI 0.905-0.953)...easy reading was 0.929...*" has been replaced by "*...was 0.928 (95% CI 0.905-0.951)… easy reading was 0.928....*"
- Under "Statistical Analysis of Multidimensional Semantic Features in English Educational Health Texts," in the first paragraph, the sentence "*…were identified as statistically significant (*P*=.005)*" has been replaced by "*...were identified as statistically significant (*P*=.01)*."
- Under "Statistical Analysis of Multidimensional Semantic Features in English Educational Health Texts," in the first paragraph, the sentence "*The odds ratio of Z7 was 0.845 (95% CI 0.751-0.951),… a difficult text was 84.5%...*" has been replaced by "*The odds ratio of Z7 was 0.86 (95% CI 0.767-0.964), …a difficult text was 86%....*"
- Under "Statistical Analysis of Multidimensional Semantic Features in English Educational Health Texts," in the first paragraph, the sentence "*The large semantic category X2 (mental actions and process) was detected as a large contributor to the cognitive accessibility of health texts (odds ratio Exp(B) 0.92, 95% CI 0.852-0.995; *P*=.04). Typical expressions included in the X2 class were English expressions related to reasoning and thinking and levels of belief or skepticism. Terms of knowledge acquisition, perception, and retrospection were included in this broad category, such as familiarize, forget, reflect, or become aware*" has been deleted.
- Under "Statistical Analysis of Multidimensional Semantic Features in English Educational Health Texts," in the second paragraph, the first sentence "*The logistic regression result (Multimedia Appendix 1) also identified 13 semantic features...*" has been replaced by "*The logistic regression result (Multimedia Appendix 1) also identified 12 semantic features....*"
- Under "Statistical Analysis of Multidimensional Semantic Features in English Educational Health Texts," in the second paragraph, the sentence "*Typical examples were B3 (medicines and medical treatment; odds ratio Exp(B) 1.042, 95% CI 1.012-1.073; *P*=.005), Z99 (out-of-dictionary words; odds ratio Exp(B) 1.01, 95% CI 1.004-1.017; *P*=.003), L2 (living creatures: animals, microorganism, virus, bacteria, etc; odds ratio Exp(B) 1.082, 95% CI 1.003-1.167; *P*=.04), and W5 (environmental terms: pollutants, carcinogens, inhalable particles, etc; odds ratio Exp(B) 2.244, 95% CI 1.11-4.538; *P*=.02)*" has been replaced by "*Typical examples were B3 (medicines and medical treatment; odds ratio Exp(B) 1.041, 95% CI 1.012-1.071; *P*=.005), Z99 (out-of-dictionary words; odds ratio Exp(B) 1.011, 95% CI 1.004-1.018; *P*=.001), L2 (living creatures: animals, microorganism, virus, bacteria, etc; odds ratio Exp(B) 1.080, 95% CI 1.005-1.162; *P*=.036), and W5 (environmental terms: pollutants, carcinogens, inhalable particles, etc.; odds ratio Exp(B) 2.441, 95% CI 1.173-5.077; *P*=.017)*."
- Under "Statistical Analysis of Multidimensional Semantic Features in English Educational Health Texts," in the second paragraph, the sentence "*For example, the relatively large odds ratios (mean 2.244, 95% CI 1.11-4.538) of W5 encompassing terms related to environmental exposure and health risks indicates that, with the increase of one word in this particular category, the odds of a health text being a difficult text over the odds of the text being an easy text for the target readers was 2.244, or in terms of percentage change, this represents an increase of 124.4% of the text from an easy text to a very difficult health reading*" has been replaced by "*For example, the relatively large odds ratios (2.441, 95% CI 1.173-5.077) of W5 encompassing terms related to environmental exposure and health risks indicates that, with the increase of one word in this particular category, the odds of a health text being a difficult text over the odds of the text being an easy text for the target readers was 2.441, or in terms of percentage change, this represents an increase of 144.1% of the text from an easy text to a very difficult health reading.*"
- Under "Statistical Analysis of Multidimensional Semantic Features in English Educational Health Texts," in the second paragraph, the sentence "*To a lesser extent, the odds ratio of 1.082 of L2 (living creatures including microorganisms) indicates that with the increase of one word in this class, the perceived difficulty level (hard-to-understand class) of the health text increased by a mean 8.2% (95% CI 0.3%-16.7%) depending on the vocabulary range of English health terms of the readers*" has been replaced by "*To a lesser extent, the odds ratio of 1.080 of L2 (living creatures including microorganisms) indicates that with the increase of one word in this class, the perceived difficulty level (hard-to-understand class) of the health text increased by a mean 8.0% (95% CI 0.5%-16.2%) depending on the vocabulary range of English health terms of the readers.*"
- Under "Statistical Analysis of Multidimensional Semantic Features in English Educational Health Texts," in the second paragraph, the sentence "*These include A2 (general or abstract terms denoting the propensity for changes, such as adapt, adjust for, conversion, and alter; odds ratio 1.057, 95% CI 1.005-1.111; *P*=.03), A7 (abstract terms of modality, such as possibility, necessity, and certainty; odds ratio 1.099, 95% CI 1.006-1.2; *P*=.04), A11 (abstract terms denoting importance, significance, noticeability, or markedness; odds ratio 1.164, 95% CI 1.003-1.351; *P*=.045)*" has been replaced by "*These include A11 (abstract terms denoting importance, significance, noticeability, or markedness; odds ratio 1.219, 95% CI 1.070-1.388; *P*=.003)*."
- Under "Statistical Analysis of Multidimensional Semantic Features in English Educational Health Texts," in the second paragraph, the sentence "*This means that with the increase of one word in the A11 class, the odds of the health text being seen as a hard-to-understand text over the text being seen as an easy text was 1.164, or an increase of 16.4%*" has been replaced by "*This means that with the increase of one unit in the A11 class, the odds of the health text being seen as a hard-to-understand text over the text being seen as an easy text was 1.219, or an increase of 21.9%*."

**Methods**

- Under "Methods," in the second paragraph, the sentences " *For a decision tree classifier, the best-point hyperparameters (Figure 1) were the maximum number of tree splits (n=22) based on Gini diversity index (minimum parent node size n=10). The observed minimal classification error of the optimized decision tree model was 0.203. For an ensemble classifier, the best-point hyperparameters (Figure 2) reached an observed minimum classification error of 0.14091. The optimized hyperparameters were the ensemble method (LogitBoost), number of learners (n=302), learning rate (0.15456), and maximum number of splits (n=9). For SVM, the best-point hyperparameters (Figure 3) were box constraint level (0.014832; kernel function: linear). The observed minimum classification error was 0.18722, lower than the optimized decision tree model (0.203) but higher than the optimized ensemble classifier (0.14091)*" have been replaced by " *For a decision tree classifier, the best-point hyperparameters (Figure 1) were the maximum number of tree splits (n=22) based on maximum deviance reduction. The observed minimal classification error of the optimized decision tree model was 0.215. For an ensemble classifier, the best-point hyperparameters (Figure 2) reached an observed minimum classification error of 0.168. The optimized hyperparameters were the ensemble method (LogitBoost), number of learners (n=210), learning rate (0.1), and maximum number of splits (n=22). For SVM, the best-point hyperparameters (Figure 3) were box constraint level (0.1), kernel function (cubic). The observed minimum classification error was 0.1944, lower than the optimized decision tree model (with a difference of 0.0206) but higher than the optimized ensemble classifier (with a difference of 0.0264).*"

**Results**

- Under "Results," in the first paragraph, the sentences "*The mean scores and SDs of the area under the operating characteristic curve (AUC), sensitivity, specificity, and accuracy were obtained through 10-fold cross-validation. The cross-validation divided the entire data set into 10 folds of equal size. In each iteration, 9 folds were used for the training data, and the remaining fold was used as the testing data. As a result, on completion of the 10-fold cross-validation, each fold was used as the testing data exactly once. We used pairwise corrected resampled *t* test to counteract the issue of multiple comparisons. As the result, the significance level was adjusted to .008 (n=6; α=.05) using Bonferroni correction*" have been replaced by "*The mean scores and standard deviations of the area under the operating characteristic curve (AUC), sensitivity, specificity, and accuracy were obtained through 5-fold cross-validation. The cross-validation divided the entire data set into 5 folds of equal size. In each iteration, 4 folds were used for the training data, and the remaining fold was used as the testing data. As a result, on completion of the 5-fold cross-validation, each fold was used as the testing data exactly once. We used paired-sample comparisons to investigate the area under the operating characteristic curve (AUC), sensitivity, specificity, and accuracy differences of four machine learning algorithms (n=6; α=.05)*."
- Under "Results," the second paragraph " *Table 2 shows that, in terms of AUC, ensemble classifier (LogitBoost), decision tree, and SVM reached statistically improved AUC over logistic regression (0.802): LogitBoost (0.97; *P*<.001), decision tree (0.924; *P*<.001), and SVM (0.8946, *P*=.002). In terms of sensitivity, only LogitBoost (0.966; *P*<.001) reached statistically significant improvement over logistic regression (0.8364), whereas decision tree (0.9122) and SVM (0.8952) had similar sensitivity as logistic regression. In terms of model specificity, LogitBoost, decision tree, and SVM all reached statistically improved specificity over logistic regression (0.7694): LogitBoost (0.972; *P*=.002), decision tree (0.9358; *P*=.003), and SVM (0.894; *P*=.004). Lastly, with regard to model overall accuracy, again, LogitBoost, decision tree, and SVM outperformed logistic regression (0.8029): LogitBoost (0.969; *P*<.001), decision tree (0.924; *P*<.001), and SVM (0.8946; *P*=.002). Comparing LogitBoost, decision tree, and SVM, the former two algorithms outperformed SVM consistently in AUC (*P*=.001), sensitivity (*P*=.007), and accuracy (*P*=.001), and LogitBoost and SVM outperformed decision tree in terms of model specificity (*P*=.003), using the adjusted .008 as the significance level of paired-sample comparisons (Bonferroni correction: n=6; α=.05). These results suggest that, when using semantic features as predictor variables, the most stable and highest-performing algorithm is ensemble classifier (LogitBoost), followed by optimized decision tree. LogitBoost, decision tree, and SVM all achieved statistically significant improvement over logistic regression in AUC, specificity, and accuracy. Decision tree and SVM did not improve over logistic regression in terms of sensitivity, but LogitBoost did. Overall, the best AUC, sensitivity, specificity, and accuracy were achieved by LogitBoost as an ensemble classifier (Figure 4)*" has been replaced by " *Table 2 shows that, in terms of AUC, ensemble classifier (LogitBoost), decision tree, and SVM reached statistically improved AUC over logistic regression (0.614): ensemble classifier (0.858; *P*=.001), decision tree (0.754; *P*=.004), and SVM (0.848, *P*=.001). In terms of sensitivity (Table 3), ensemble classifier (0.787, *P*=.020), decision tree (0.7174, *P*=.036), and SVM (0.783; *P*<.001) reached statistically significant improvement over logistic regression (0.6282). In terms of model specificity (Table 4), ensemble classifier, decision tree, and SVM all reached statistically improved specificity over logistic regression (0.5724): ensemble classifier (0.813; *P*=.001), decision tree (0.7424; *P*=.009), and SVM (0.791; *P*=.007). Lastly, with regard to model overall accuracy (Table 5), again, LogitBoost, decision tree, and SVM outperformed logistic regression (0.601): ensemble classifier (0.802; *P*=.001), decision tree (0.732; *P*=.003), and SVM (0.786; *P*=.001). Comparing SVM, ensemble classifier and decision tree, the former two algorithms outperformed decision tree consistently in AUC (*P*=.001 and *P*<.001, respectively), and accuracy (*P*=.022 and *P*=.001, respectively). Only ensemble classifier outperformed decision tree significantly in terms of model sensitivity (*P*=.024), and specificity (*P*=.010), using the paired-sample comparisons (n=6; α=.05). These results suggest that, when using semantic features as predictor variables, the most stable and highest-performing algorithm is ensemble classifier (LogitBoost), followed by SVM. Ensemble classifier, decision tree, and SVM all achieved statistically significant improvement over logistic regression in AUC, specificity, sensitivity, and accuracy. SVM did not improve significantly over decision tree in terms of sensitivity and specificity, but ensemble classifier did. Overall, the best AUC, sensitivity, specificity, and accuracy were achieved by LogitBoost as an ensemble classifier (Figure 4)*."

**Discussion**

- Under "Principal Findings," in the second paragraph, the sentence "*…(measured in pairwise resampled *t* tests, with *P* value adjusted to .008 using Bonferroni correction)*" has been replaced by "*…(measured in pairwise resampled *t* tests)*."
- Under "Principal Findings," in the last paragraph, the sentence "*…or those requiring higher cognitive abilities, such as assessing the propensity for changes and expressions of modality describing possibility, necessity, and certainty of health events and situations*" has been replaced by "*…or those requiring higher cognitive abilities, such as abstract terms denoting importance, significance, noticeability or markedness of health events and situations*."

**Authors' Contributions**

In the originally published paper, the following "Authors' Contributions" section was not included.

MJ and TH were responsible for overall research design; MJ was responsible for paper writing and revision, and YL was responsible for formal analysis and data curation.

**Multimedia Appendices**

The information presented in the Multimedia Appendix 1 entitled "Variables in the logistic regression of health text understandability membership" has been updated. The originally published Multimedia Appendix 1 is in Multimedia Appendix 2.

**Figures and Tables**

Figures 1-4 have been replaced and can be viewed below. The originally published Figures 1-4 are in Multimedia Appendix 3. Tables 1-5 have been updated and can be viewed below. The originally published Tables 1-5 are in Multimedia Appendix 4.

**Figure 1:**
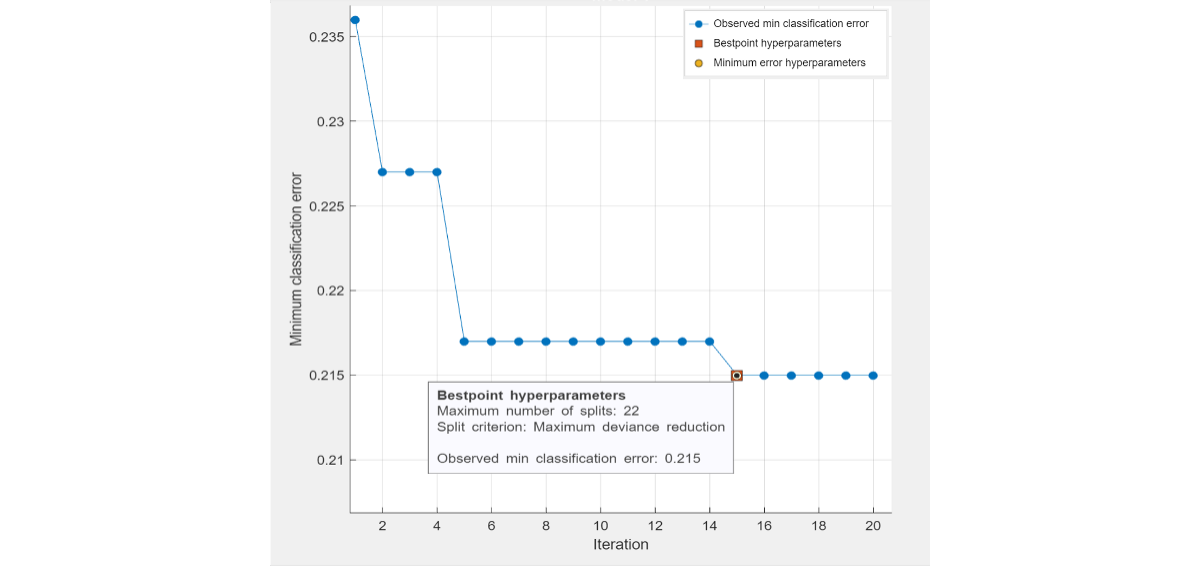
Hyperparameter tuning (decision tree).

**Figure 2:**
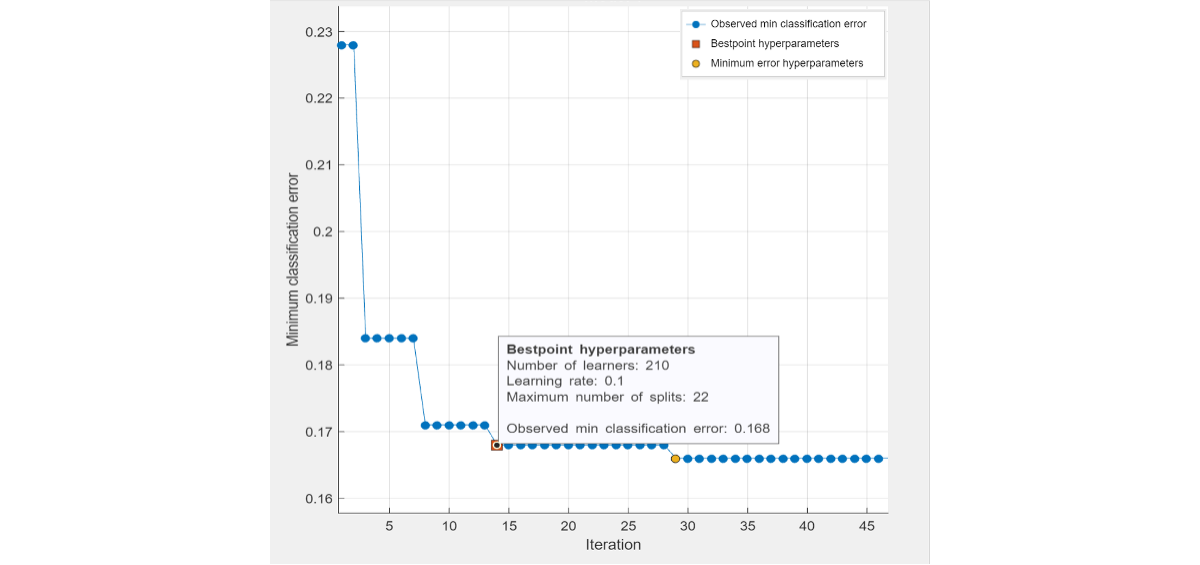
Hyperparameter tuning (ensemble classifier).

**Figure 3:**
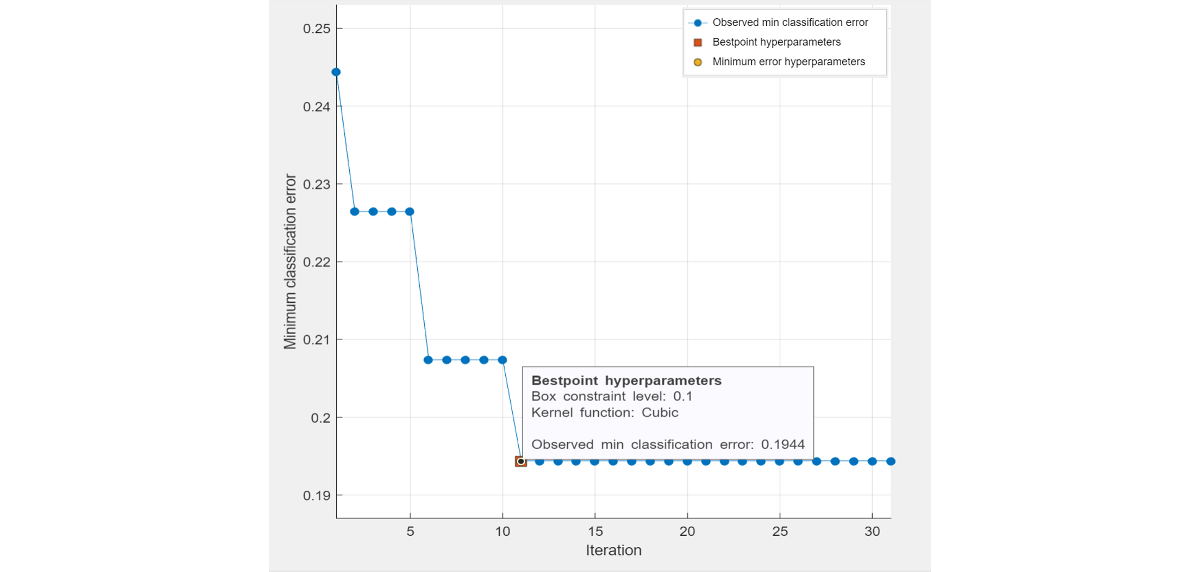
Hyperparameter tuning (support vector machine).

**Figure 4:**
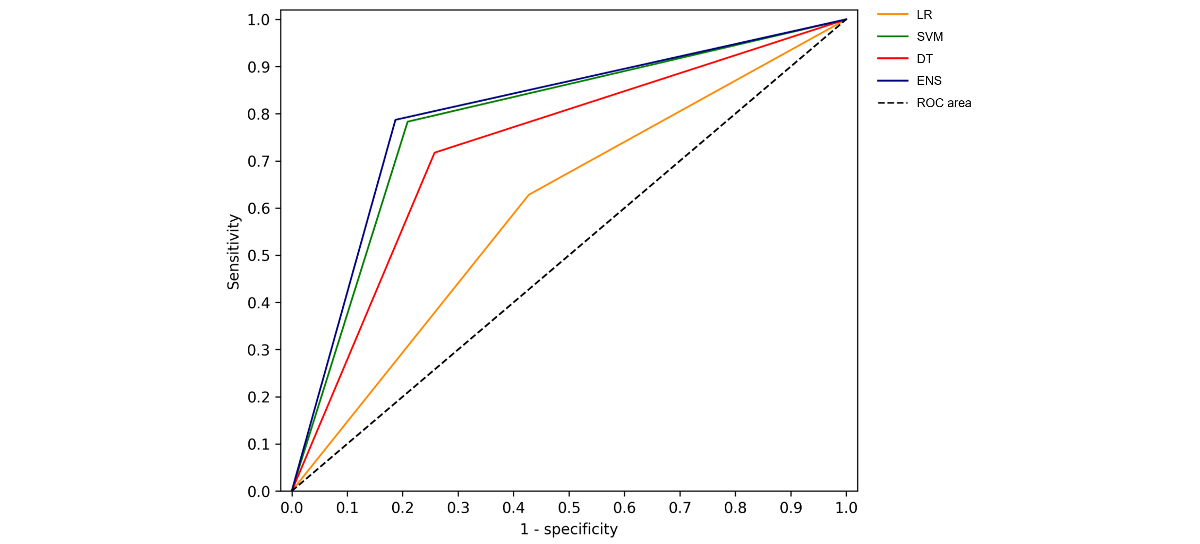
Mean receiver operating characteristic (ROC) curve for machine learning algorithms. LR: logistic regression; SVM: support vector machine; DT: decision tree; ENS: ensemble classifier (LogitBoost); ROC: receiver operating characteristic.

**Table 1:** Performance of the machine learning models using multidimensional semantic features as predictors.

Algorithm	AUC^a^, mean (SD)	Sensitivity, mean (SD)	Specificity, mean (SD)	Accuracy, mean (SD)
LR^b^	0.614 (0.0554)	0.6282 (0.0597)	0.5724 (0.0733)	0.6010 (0.0523)
SVM^c^	0.848 (0.0172)	0.7830 (0.0368)	0.7910 (0.0420)	0.7860 (0.0153)
DT^d^	0.754 (0.0377)	0.7174 (0.0719)	0.7424 (0.0589)	0.732 (0.0317)
ENS^e^	0.858 (0.041)	0.787 (0.057)	0.813 (0.046)	0.802 (0.032)

^a^AUC: area under the operating characteristic curve.

^b^LR: logistic regression.

^c^SVM: support vector machine.

^d^DT: decision tree.

^e^ENS: ensemble classifier (LogitBoost).

**Table 2:** Pairwise corrected resampled *t* test of area under the curve differences (using multidimensional semantic features as predictor variables).

Pairs	Mean difference (SD)	Standard error mean	95% CI	*t* test (*df*)	*P* value
LR^a^ vs SVM^b^	–0.2340 (0.0669)	0.0299	–0.3171 to –0.1509	–7.817 (4)	.001
LR vs DT^c^	–0.1460 (0.0551)	0.0246	–0.2144 to –0.0777	–5.931 (4)	.004
LR vs ENS^d^	–0.2440 (0.0564)	0.0252	–0.3140 to –0.1740	–9.675 (4)	.001
SVM vs DT	0.0880 (0.0192)	0.0086	–0.0641 to 0.1119	10.230 (4)	.001
SVM vs ENS	–0.0100 (0.0374)	0.0167	–0.0565 to –0.0365	–0.598 (4)	.582
DT vs ENS	–0.0980 (0.0192)	0.0086	–0.1219 to –0.0741	–11.392 (4)	<.001

^a^LR: logistic regression.

^b^SVM: support vector machine.

^c^DT: decision tree.

^d^ENS: ensemble classifier (LogitBoost).

**Table 3:** Pairwise corrected resampled *t* test of sensitivity differences (using multidimensional semantic features as predictor variables).

Pairs	Mean difference (SD)	Standard error mean	95% CI	*t* test (*df*)	*P* value
LR^a^ vs SVM^b^	–0.1548 (0.0303)	0.0135	–0.1924 to –0.1172	–11.429 (4)	<.001
LR vs DT^c^	–0.1002 (0.0720)	0.0322	–0.1896 to –0.0108	–3.111 (4)	.036
LR vs ENS^d^	–0.1588 (0.0945)	0.0423	–0.2761 to –0.0414	–3.756 (4)	.020
SVM vs DT	0.0546 (0.0697)	0.0312	–0.0319 to 0.1411	1.752 (4)	.155
SVM vs ENS	–0.0040 (0.0855)	0.0382	–0.1102 to –0.1022	–0.105 (4)	.922
DT vs ENS	–0.0586 (0.0371)	0.0166	–0.1046 to –0.0126	–3.535 (4)	.024

^a^LR: logistic regression.

^b^SVM: support vector machine.

^c^DT: decision tree.

^d^ENS: ensemble classifier (LogitBoost).

**Table 4:** Pairwise corrected resampled *t* test of specificity differences (using multidimensional semantic features as predictor variables).

Pairs	Mean difference (SD)	Standard error mean	95% CI	*t* test (*df*)	*P* value
LR^a^ vs SVM^b^	–0.2186 (0.0968)	0.0433	–0.3389 to –0.0984	–5.047 (4)	.007
LR vs DT^c^	–0.1720 (0.0822)	0.0368	–0.2741 to –0.0699	–4.679 (4)	.009
LR vs ENS^d^	–0.2410 (0.0677)	0.0303	–0.3251 to –0.1569	–7.959 (4)	.001
SVM vs DT	0.0466 (0.1059)	0.0474	–0.0849 to 0.1781	0.984 (4)	.381
SVM vs ENS	–0.0224 (0.0918)	0.0411	–0.1364 to –0.0916	–0.545 (4)	.614
DT vs ENS	–0.0690 (0.0334)	0.0149	–0.1105 to –0.0275	–4.619 (4)	.010

^a^LR: logistic regression.

^b^SVM: support vector machine.

^c^DT: decision tree.

^d^ENS: ensemble classifier (LogitBoost).

**Table 5:** Pairwise corrected resampled *t* test of accuracy differences (using multidimensional semantic features as predictor variables).

Pairs	Mean difference (SD)	Standard error mean	95% CI	*t* test (*df*)	*P* value
LR^a^ vs SVM^b^	–0.1850 (0.0507)	0.0227	–0.2480 to –0.1220	–8.152 (4)	.001
LR vs DT^c^	–0.1370 (0.0482)	0.0215	–0.1968 to –0.0771	–6.360 (4)	.003
LR vs ENS^d^	–0.2010 (0.0549)	0.0246	–0.2692 to –0.1328	–8.182 (4)	.001
SVM vs DT	0.0480 (0.0295)	0.0132	0.0114 to 0.0846	3.639 (4)	.022
SVM vs ENS	–0.0160 (0.0366)	0.0164	–0.0615 to 0.0295	–0.976 (4)	.384
DT vs ENS	–0.0640 (0.0148)	0.0066	–0.0823 to –0.0457	–9.704 (4)	.001

^a^LR: logistic regression.

^b^SVM: support vector machine.

^c^DT: decision tree.

^d^ENS: ensemble classifier (LogitBoost).

The authors confirm that the results and conclusions of the corrected data are consistent with those in the originally published version.

These corrections will appear in the online version of the paper on the JMIR website on September 21, 2021, together with the publication of this correction notice. Because this was made after submission to PubMed, PubMed Central, and other full-text repositories, the corrected article has also been resubmitted to those repositories.

## Appendix

Multimedia Appendix 1 Variables in the logistic regression of health text understandability membership.

Multimedia Appendix 2 Originally published Multimedia Appendix 1.

Multimedia Appendix 3 Originally published Figures 1-4.

Multimedia Appendix 4 Originally published Tables 1-5.

