# Supplementary material for: Predicting Health Material Accessibility: Development of Machine Learning Algorithms
Source: JMIR Med Inform. 2021 Sep 1;9(9):e29175. doi: 10.2196/29175 (PMC8444043; doi:10.2196/29175)
Supplement: Multimedia Appendix 1 [file medinform_v9i9e29175_app1.docx]

Multimedia Appendix 1. Variables in the Logistic Regression of Health Text Understandability Membership.

|  | B | S.E. | Wald | df | Sig. | Exp(B) | 95% C.I. for EXP(B) | |
| --- | --- | --- | --- | --- | --- | --- | --- | --- |
|  |  |  |  |  |  |  | Lower | Upper |
| M4 | -0.411 | 0.134 | 9.409 | 1 | 0.002 | 0.663 | 0.510 | 0.862 |
| G2 | -0.351 | 0.123 | 8.089 | 1 | 0.004 | 0.704 | 0.553 | 0.897 |
| E4 | -0.266 | 0.132 | 4.027 | 1 | 0.045 | 0.767 | 0.592 | 0.994 |
| G1 | -0.223 | 0.100 | 4.981 | 1 | 0.026 | 0.800 | 0.658 | 0.973 |
| L3 | -0.177 | 0.057 | 9.652 | 1 | 0.002 | 0.838 | 0.750 | 0.937 |
| A4 | -0.169 | 0.050 | 11.325 | 1 | 0.001 | 0.845 | 0.766 | 0.932 |
| N6 | -0.159 | 0.052 | 9.482 | 1 | 0.002 | 0.853 | 0.771 | 0.944 |
| Z7 | -0.151 | 0.058 | 6.679 | 1 | 0.010 | 0.860 | 0.767 | 0.964 |
| S8 | -0.103 | 0.039 | 6.792 | 1 | 0.009 | 0.902 | 0.835 | 0.975 |
| O2 | -0.100 | 0.033 | 9.013 | 1 | 0.003 | 0.905 | 0.848 | 0.966 |
| A9 | -0.096 | 0.044 | 4.866 | 1 | 0.027 | 0.908 | 0.834 | 0.989 |
| Z3 | -0.083 | 0.036 | 5.305 | 1 | 0.021 | 0.920 | 0.857 | 0.988 |
| Z8 | -0.075 | 0.013 | 35.022 | 1 | 0.000 | 0.928 | 0.905 | 0.951 |
| F1 | -0.035 | 0.016 | 4.881 | 1 | 0.027 | 0.965 | 0.936 | 0.996 |
| Z99 | 0.011 | 0.003 | 10.557 | 1 | 0.001 | 1.011 | 1.004 | 1.018 |
| Z5 | 0.030 | 0.008 | 16.490 | 1 | 0.000 | 1.031 | 1.016 | 1.046 |
| B3 | 0.040 | 0.014 | 7.757 | 1 | 0.005 | 1.041 | 1.012 | 1.071 |
| L2 | 0.077 | 0.037 | 4.378 | 1 | 0.036 | 1.080 | 1.005 | 1.162 |
| F3 | 0.163 | 0.063 | 6.597 | 1 | 0.010 | 1.177 | 1.039 | 1.332 |
| X7 | 0.166 | 0.048 | 11.930 | 1 | 0.001 | 1.181 | 1.075 | 1.297 |
| A11 | 0.198 | 0.066 | 8.876 | 1 | 0.003 | 1.219 | 1.070 | 1.388 |
| A10 | 0.228 | 0.073 | 9.774 | 1 | 0.002 | 1.256 | 1.089 | 1.448 |
| W3 | 0.413 | 0.207 | 3.981 | 1 | 0.046 | 1.512 | 1.007 | 2.270 |
| N2 | 0.534 | 0.227 | 5.540 | 1 | 0.019 | 1.705 | 1.093 | 2.659 |
| W1 | 0.860 | 0.399 | 4.657 | 1 | 0.031 | 2.364 | 1.082 | 5.164 |
| W5 | 0.892 | 0.374 | 5.702 | 1 | 0.017 | 2.441 | 1.173 | 5.077 |
|  |  |  |  |  |  |  |  |  |
| S5 | -0.114 | 0.059 | 3.729 | 1 | 0.053 | 0.892 | 0.795 | 1.002 |
| O4 | -0.055 | 0.029 | 3.637 | 1 | 0.057 | 0.946 | 0.894 | 1.002 |
| A3 | -0.070 | 0.037 | 3.615 | 1 | 0.057 | 0.933 | 0.868 | 1.002 |
| Z4 | -0.155 | 0.084 | 3.403 | 1 | 0.065 | 0.857 | 0.727 | 1.010 |
| W4 | 0.336 | 0.184 | 3.347 | 1 | 0.067 | 1.399 | 0.976 | 2.005 |
| X2 | -0.068 | 0.038 | 3.198 | 1 | 0.074 | 0.935 | 0.868 | 1.007 |
| H4 | -0.324 | 0.181 | 3.193 | 1 | 0.074 | 0.723 | 0.507 | 1.032 |
| A2 | 0.043 | 0.025 | 3.051 | 1 | 0.081 | 1.044 | 0.995 | 1.095 |
| T2 | 0.104 | 0.060 | 3.020 | 1 | 0.082 | 1.110 | 0.987 | 1.248 |
| F2 | -0.094 | 0.054 | 3.010 | 1 | 0.083 | 0.911 | 0.819 | 1.012 |
| N3 | -0.046 | 0.027 | 2.898 | 1 | 0.089 | 0.955 | 0.906 | 1.007 |
| S4 | -0.103 | 0.061 | 2.875 | 1 | 0.090 | 0.902 | 0.800 | 1.016 |
| A7 | 0.071 | 0.043 | 2.683 | 1 | 0.101 | 1.074 | 0.986 | 1.169 |
| E3 | 0.062 | 0.038 | 2.647 | 1 | 0.104 | 1.064 | 0.987 | 1.147 |
| A15 | 0.099 | 0.061 | 2.572 | 1 | 0.109 | 1.104 | 0.978 | 1.245 |
| Z6 | 0.100 | 0.065 | 2.361 | 1 | 0.124 | 1.105 | 0.973 | 1.254 |
| T3 | -0.041 | 0.027 | 2.304 | 1 | 0.129 | 0.959 | 0.910 | 1.012 |
| E5 | 0.367 | 0.244 | 2.261 | 1 | 0.133 | 1.443 | 0.895 | 2.328 |
| L1 | -0.091 | 0.060 | 2.260 | 1 | 0.133 | 0.913 | 0.811 | 1.028 |
| A8 | -0.474 | 0.316 | 2.247 | 1 | 0.134 | 0.623 | 0.335 | 1.157 |
| X6 | 0.337 | 0.227 | 2.199 | 1 | 0.138 | 1.400 | 0.897 | 2.184 |
| I1 | -0.127 | 0.088 | 2.048 | 1 | 0.152 | 0.881 | 0.741 | 1.048 |
| S1 | -0.077 | 0.055 | 2.016 | 1 | 0.156 | 0.925 | 0.832 | 1.030 |
| E2 | -0.191 | 0.142 | 1.792 | 1 | 0.181 | 0.826 | 0.625 | 1.093 |
| K4 | 0.505 | 0.393 | 1.651 | 1 | 0.199 | 1.658 | 0.767 | 3.583 |
| X5 | 0.056 | 0.046 | 1.523 | 1 | 0.217 | 1.058 | 0.967 | 1.157 |
| Y1 | 0.125 | 0.103 | 1.479 | 1 | 0.224 | 1.134 | 0.926 | 1.387 |
| S2 | 0.024 | 0.020 | 1.476 | 1 | 0.224 | 1.025 | 0.985 | 1.065 |
| X4 | 0.093 | 0.078 | 1.443 | 1 | 0.230 | 1.098 | 0.943 | 1.278 |
| B5 | 0.044 | 0.037 | 1.417 | 1 | 0.234 | 1.045 | 0.972 | 1.123 |
| A5 | 0.051 | 0.043 | 1.405 | 1 | 0.236 | 1.053 | 0.967 | 1.146 |
| B1 | 0.013 | 0.011 | 1.390 | 1 | 0.238 | 1.013 | 0.992 | 1.035 |
| K3 | 0.270 | 0.240 | 1.265 | 1 | 0.261 | 1.310 | 0.818 | 2.099 |
| Q3 | -0.071 | 0.066 | 1.167 | 1 | 0.280 | 0.931 | 0.818 | 1.060 |
| K2 | -0.467 | 0.432 | 1.167 | 1 | 0.280 | 0.627 | 0.269 | 1.463 |
| Z1 | -0.029 | 0.027 | 1.137 | 1 | 0.286 | 0.972 | 0.922 | 1.024 |
| M3 | 0.089 | 0.084 | 1.132 | 1 | 0.287 | 1.093 | 0.928 | 1.289 |
| B2 | 0.010 | 0.010 | 1.038 | 1 | 0.308 | 1.010 | 0.991 | 1.029 |
| S9 | -0.119 | 0.118 | 1.016 | 1 | 0.314 | 0.887 | 0.704 | 1.119 |
| O3 | 0.293 | 0.299 | 0.960 | 1 | 0.327 | 1.340 | 0.746 | 2.406 |
| M5 | 0.063 | 0.065 | 0.952 | 1 | 0.329 | 1.065 | 0.938 | 1.210 |
| X8 | 0.143 | 0.153 | 0.878 | 1 | 0.349 | 1.154 | 0.855 | 1.558 |
| A6 | -0.030 | 0.035 | 0.719 | 1 | 0.397 | 0.971 | 0.906 | 1.040 |
| B4 | 0.028 | 0.035 | 0.667 | 1 | 0.414 | 1.029 | 0.961 | 1.101 |
| P1 | 0.036 | 0.049 | 0.536 | 1 | 0.464 | 1.037 | 0.942 | 1.141 |
| H2 | 0.096 | 0.132 | 0.525 | 1 | 0.469 | 1.100 | 0.850 | 1.425 |
| M6 | -0.032 | 0.045 | 0.513 | 1 | 0.474 | 0.969 | 0.887 | 1.057 |
| M2 | -0.048 | 0.068 | 0.508 | 1 | 0.476 | 0.953 | 0.834 | 1.088 |
| I3 | -0.034 | 0.047 | 0.505 | 1 | 0.477 | 0.967 | 0.881 | 1.061 |
| S6 | -0.044 | 0.063 | 0.495 | 1 | 0.482 | 0.957 | 0.846 | 1.082 |
| I2 | 0.069 | 0.098 | 0.495 | 1 | 0.482 | 1.072 | 0.884 | 1.299 |
| A13 | 0.036 | 0.051 | 0.491 | 1 | 0.483 | 1.036 | 0.938 | 1.145 |
| S7 | 0.039 | 0.057 | 0.471 | 1 | 0.492 | 1.040 | 0.929 | 1.164 |
| A14 | 0.118 | 0.179 | 0.436 | 1 | 0.509 | 1.126 | 0.792 | 1.599 |
| X9 | 0.038 | 0.064 | 0.363 | 1 | 0.547 | 1.039 | 0.917 | 1.178 |
| Q1 | 0.028 | 0.048 | 0.331 | 1 | 0.565 | 1.028 | 0.936 | 1.129 |
| A1 | 0.014 | 0.026 | 0.310 | 1 | 0.578 | 1.015 | 0.964 | 1.067 |
| A12 | -0.044 | 0.080 | 0.302 | 1 | 0.583 | 0.957 | 0.817 | 1.120 |
| E6 | -0.021 | 0.044 | 0.230 | 1 | 0.631 | 0.979 | 0.898 | 1.068 |
| Y2 | 0.048 | 0.100 | 0.228 | 1 | 0.633 | 1.049 | 0.863 | 1.275 |
| M1 | -0.024 | 0.053 | 0.212 | 1 | 0.645 | 0.976 | 0.880 | 1.082 |
| K6 | -0.158 | 0.379 | 0.173 | 1 | 0.677 | 0.854 | 0.406 | 1.796 |
| C1 | 0.049 | 0.120 | 0.169 | 1 | 0.681 | 1.051 | 0.830 | 1.329 |
| F4 | -0.050 | 0.128 | 0.151 | 1 | 0.698 | 0.952 | 0.741 | 1.222 |
| T1 | 0.007 | 0.019 | 0.147 | 1 | 0.701 | 1.007 | 0.971 | 1.045 |
| H5 | -0.044 | 0.116 | 0.147 | 1 | 0.701 | 0.957 | 0.762 | 1.201 |
| I4 | -0.168 | 0.453 | 0.137 | 1 | 0.711 | 0.846 | 0.348 | 2.054 |
| G3 | -0.125 | 0.352 | 0.126 | 1 | 0.723 | 0.883 | 0.443 | 1.758 |
| K1 | 0.076 | 0.238 | 0.101 | 1 | 0.751 | 1.079 | 0.676 | 1.721 |
| H3 | -0.154 | 0.515 | 0.089 | 1 | 0.765 | 0.857 | 0.313 | 2.351 |
| Q2 | -0.008 | 0.029 | 0.080 | 1 | 0.777 | 0.992 | 0.937 | 1.050 |
| X1 | -0.016 | 0.060 | 0.074 | 1 | 0.785 | 0.984 | 0.875 | 1.106 |
| S3 | -0.013 | 0.052 | 0.061 | 1 | 0.804 | 0.987 | 0.891 | 1.093 |
| X3 | -0.009 | 0.038 | 0.056 | 1 | 0.812 | 0.991 | 0.919 | 1.068 |
| M8 | 0.041 | 0.178 | 0.052 | 1 | 0.820 | 1.041 | 0.734 | 1.477 |
| Z2 | -0.011 | 0.048 | 0.049 | 1 | 0.825 | 0.989 | 0.900 | 1.087 |
| N5 | 0.005 | 0.022 | 0.046 | 1 | 0.831 | 1.005 | 0.963 | 1.049 |
| H1 | -0.025 | 0.123 | 0.043 | 1 | 0.836 | 0.975 | 0.766 | 1.241 |
| K5 | -0.014 | 0.073 | 0.034 | 1 | 0.853 | 0.987 | 0.855 | 1.138 |
| M7 | 0.008 | 0.063 | 0.017 | 1 | 0.896 | 1.008 | 0.891 | 1.141 |
| T4 | 0.017 | 0.133 | 0.017 | 1 | 0.896 | 1.018 | 0.783 | 1.322 |
| O1 | 0.003 | 0.023 | 0.015 | 1 | 0.901 | 1.003 | 0.959 | 1.048 |
| N4 | 0.008 | 0.069 | 0.012 | 1 | 0.912 | 1.008 | 0.881 | 1.152 |
| E1 | 0.018 | 0.201 | 0.008 | 1 | 0.930 | 1.018 | 0.686 | 1.509 |
| W2 | 0.004 | 0.175 | 0.000 | 1 | 0.982 | 1.004 | 0.713 | 1.414 |
| Q4 | 0.001 | 0.094 | 0.000 | 1 | 0.988 | 1.001 | 0.833 | 1.204 |
| N1 | 0.000 | 0.030 | 0.000 | 1 | 0.996 | 1.000 | 0.943 | 1.060 |
| Constant | -0.157 | 0.200 | 0.618 | 1 | 0.432 | 0.855 |  |  |

Method = Backward Stepwise (Conditional)
