# Supplementary material for: Correction: Predicting Health Material Accessibility: Development of Machine Learning Algorithms
Source: JMIR Med Inform. 2021 Sep 21;9(9):e33385. doi: 10.2196/33385 (PMC8493462; doi:10.2196/33385)
Supplement: Multimedia Appendix 2 [file medinform_v9i9e33385_app2.docx]

Multimedia Appendix 2: Originally published Multimedia Appendix 1.

|  | B | S.E. | Wald | df | Sig. | Exp(B) | 95% C.I. for EXP(B) | |
| --- | --- | --- | --- | --- | --- | --- | --- | --- |
|  |  |  |  |  |  |  | Lower | Upper |
| M4 | -0.48 | 0.138 | 12.025 | 1 | 0.001 | 0.619 | 0.472 | 0.812 |
| G2 | -0.333 | 0.126 | 7.036 | 1 | 0.008 | 0.717 | 0.56 | 0.917 |
| E4 | -0.302 | 0.13 | 5.389 | 1 | 0.02 | 0.739 | 0.573 | 0.954 |
| A4 | -0.174 | 0.051 | 11.771 | 1 | 0.001 | 0.84 | 0.761 | 0.928 |
| Z7 | -0.169 | 0.06 | 7.831 | 1 | 0.005 | 0.845 | 0.751 | 0.951 |
| N6 | -0.156 | 0.055 | 8.214 | 1 | 0.004 | 0.855 | 0.769 | 0.952 |
| L3 | -0.147 | 0.064 | 5.285 | 1 | 0.022 | 0.863 | 0.761 | 0.979 |
| A9 | -0.128 | 0.045 | 7.99 | 1 | 0.005 | 0.88 | 0.805 | 0.962 |
| S1 | -0.113 | 0.057 | 3.896 | 1 | 0.048 | 0.893 | 0.799 | 0.999 |
| O2 | -0.102 | 0.034 | 8.94 | 1 | 0.003 | 0.903 | 0.845 | 0.965 |
| S8 | -0.098 | 0.04 | 6.001 | 1 | 0.014 | 0.907 | 0.838 | 0.981 |
| O4 | -0.084 | 0.032 | 6.761 | 1 | 0.009 | 0.92 | 0.863 | 0.98 |
| X2 | -0.083 | 0.04 | 4.399 | 1 | 0.036 | 0.92 | 0.852 | 0.995 |
| Z8 | -0.074 | 0.013 | 32.49 | 1 | 0 | 0.929 | 0.905 | 0.953 |
| Z3 | -0.073 | 0.037 | 3.901 | 1 | 0.048 | 0.93 | 0.865 | 0.999 |
| F1 | -0.034 | 0.016 | 4.249 | 1 | 0.039 | 0.967 | 0.936 | 0.998 |
| Z99 | 0.01 | 0.003 | 9.056 | 1 | 0.003 | 1.01 | 1.004 | 1.017 |
| Z5 | 0.032 | 0.008 | 18.031 | 1 | 0 | 1.033 | 1.018 | 1.048 |
| B3 | 0.041 | 0.015 | 7.717 | 1 | 0.005 | 1.042 | 1.012 | 1.073 |
| A2 | 0.055 | 0.026 | 4.71 | 1 | 0.03 | 1.057 | 1.005 | 1.111 |
| L2 | 0.079 | 0.038 | 4.174 | 1 | 0.041 | 1.082 | 1.003 | 1.167 |
| A7 | 0.094 | 0.045 | 4.372 | 1 | 0.037 | 1.099 | 1.006 | 1.2 |
| A11 | 0.152 | 0.076 | 4.004 | 1 | 0.045 | 1.164 | 1.003 | 1.351 |
| F3 | 0.154 | 0.071 | 4.772 | 1 | 0.029 | 1.167 | 1.016 | 1.34 |
| X7 | 0.171 | 0.048 | 12.951 | 1 | 0 | 1.187 | 1.081 | 1.303 |
| A10 | 0.221 | 0.074 | 8.959 | 1 | 0.003 | 1.247 | 1.079 | 1.44 |
| W3 | 0.541 | 0.219 | 6.085 | 1 | 0.014 | 1.717 | 1.118 | 2.639 |
| N2 | 0.597 | 0.233 | 6.582 | 1 | 0.01 | 1.818 | 1.151 | 2.869 |
| W5 | 0.808 | 0.359 | 5.065 | 1 | 0.024 | 2.244 | 1.11 | 4.538 |
|  |  |  |  |  |  |  |  |  |
| A3 | -0.07 | 0.037 | 3.61 | 1 | 0.057 | 0.932 | 0.868 | 1.002 |
| E5 | 0.475 | 0.249 | 3.628 | 1 | 0.057 | 1.607 | 0.986 | 2.62 |
| F2 | -0.147 | 0.078 | 3.52 | 1 | 0.061 | 0.863 | 0.74 | 1.007 |
| B5 | 0.069 | 0.037 | 3.37 | 1 | 0.066 | 1.071 | 0.995 | 1.152 |
| G1 | -0.195 | 0.107 | 3.353 | 1 | 0.067 | 0.823 | 0.667 | 1.014 |
| A15 | 0.111 | 0.062 | 3.204 | 1 | 0.073 | 1.118 | 0.989 | 1.262 |
| S5 | -0.098 | 0.056 | 2.999 | 1 | 0.083 | 0.907 | 0.812 | 1.013 |
| Z6 | 0.116 | 0.067 | 2.993 | 1 | 0.084 | 1.123 | 0.985 | 1.28 |
| N3 | -0.047 | 0.027 | 2.902 | 1 | 0.088 | 0.954 | 0.904 | 1.007 |
| Z4 | -0.139 | 0.086 | 2.597 | 1 | 0.107 | 0.87 | 0.735 | 1.031 |
| T3 | -0.045 | 0.029 | 2.493 | 1 | 0.114 | 0.956 | 0.904 | 1.011 |
| E2 | -0.233 | 0.153 | 2.306 | 1 | 0.129 | 0.792 | 0.587 | 1.07 |
| X6 | 0.348 | 0.231 | 2.275 | 1 | 0.131 | 1.416 | 0.901 | 2.225 |
| M3 | 0.117 | 0.078 | 2.242 | 1 | 0.134 | 1.124 | 0.964 | 1.31 |
| X8 | 0.234 | 0.156 | 2.251 | 1 | 0.134 | 1.263 | 0.931 | 1.714 |
| S6 | -0.095 | 0.066 | 2.038 | 1 | 0.153 | 0.91 | 0.799 | 1.036 |
| Y1 | 0.14 | 0.101 | 1.915 | 1 | 0.166 | 1.15 | 0.944 | 1.401 |
| B2 | 0.014 | 0.01 | 1.894 | 1 | 0.169 | 1.014 | 0.994 | 1.033 |
| E3 | 0.054 | 0.04 | 1.827 | 1 | 0.176 | 1.056 | 0.976 | 1.142 |
| X4 | 0.106 | 0.079 | 1.82 | 1 | 0.177 | 1.112 | 0.953 | 1.297 |
| A8 | -0.398 | 0.299 | 1.769 | 1 | 0.183 | 0.672 | 0.374 | 1.207 |
| I1 | -0.116 | 0.087 | 1.749 | 1 | 0.186 | 0.891 | 0.75 | 1.057 |
| K2 | -0.55 | 0.416 | 1.749 | 1 | 0.186 | 0.577 | 0.255 | 1.304 |
| L1 | -0.077 | 0.061 | 1.608 | 1 | 0.205 | 0.926 | 0.821 | 1.043 |
| A5 | 0.053 | 0.044 | 1.399 | 1 | 0.237 | 1.054 | 0.966 | 1.15 |
| M5 | 0.076 | 0.066 | 1.354 | 1 | 0.245 | 1.079 | 0.949 | 1.228 |
| H3 | -0.464 | 0.402 | 1.337 | 1 | 0.248 | 0.629 | 0.286 | 1.381 |
| O3 | 0.352 | 0.31 | 1.29 | 1 | 0.256 | 1.423 | 0.774 | 2.613 |
| S4 | -0.073 | 0.065 | 1.272 | 1 | 0.259 | 0.93 | 0.819 | 1.055 |
| T2 | 0.067 | 0.061 | 1.205 | 1 | 0.272 | 1.069 | 0.949 | 1.205 |
| K4 | 0.4 | 0.366 | 1.197 | 1 | 0.274 | 1.492 | 0.728 | 3.057 |
| K3 | 0.241 | 0.225 | 1.152 | 1 | 0.283 | 1.273 | 0.82 | 1.976 |
| H2 | 0.138 | 0.135 | 1.045 | 1 | 0.307 | 1.148 | 0.881 | 1.497 |
| M2 | -0.073 | 0.073 | 0.998 | 1 | 0.318 | 0.929 | 0.805 | 1.073 |
| X5 | 0.046 | 0.047 | 0.972 | 1 | 0.324 | 1.047 | 0.955 | 1.148 |
| B4 | 0.032 | 0.034 | 0.878 | 1 | 0.349 | 1.033 | 0.966 | 1.104 |
| B1 | 0.01 | 0.011 | 0.83 | 1 | 0.362 | 1.01 | 0.988 | 1.032 |
| H4 | -0.165 | 0.186 | 0.786 | 1 | 0.375 | 0.848 | 0.589 | 1.221 |
| Z1 | -0.024 | 0.027 | 0.773 | 1 | 0.379 | 0.976 | 0.925 | 1.03 |
| Q3 | -0.063 | 0.072 | 0.761 | 1 | 0.383 | 0.939 | 0.815 | 1.082 |
| M1 | -0.044 | 0.053 | 0.692 | 1 | 0.406 | 0.957 | 0.863 | 1.061 |
| S2 | 0.017 | 0.021 | 0.638 | 1 | 0.424 | 1.017 | 0.976 | 1.059 |
| K1 | 0.19 | 0.241 | 0.625 | 1 | 0.429 | 1.21 | 0.755 | 1.938 |
| W1 | 0.332 | 0.421 | 0.621 | 1 | 0.431 | 1.393 | 0.611 | 3.178 |
| O1 | 0.018 | 0.023 | 0.616 | 1 | 0.433 | 1.018 | 0.973 | 1.066 |
| S9 | -0.087 | 0.117 | 0.557 | 1 | 0.455 | 0.916 | 0.729 | 1.152 |
| A13 | 0.038 | 0.052 | 0.541 | 1 | 0.462 | 1.039 | 0.939 | 1.149 |
| F4 | -0.096 | 0.135 | 0.504 | 1 | 0.478 | 0.909 | 0.697 | 1.184 |
| Y2 | 0.07 | 0.101 | 0.487 | 1 | 0.485 | 1.073 | 0.881 | 1.307 |
| A1 | 0.018 | 0.026 | 0.463 | 1 | 0.496 | 1.018 | 0.967 | 1.072 |
| H5 | -0.084 | 0.134 | 0.393 | 1 | 0.531 | 0.92 | 0.707 | 1.195 |
| M6 | -0.028 | 0.045 | 0.38 | 1 | 0.538 | 0.973 | 0.89 | 1.063 |
| P1 | 0.029 | 0.05 | 0.342 | 1 | 0.559 | 1.03 | 0.933 | 1.136 |
| Q1 | 0.027 | 0.048 | 0.323 | 1 | 0.57 | 1.028 | 0.935 | 1.129 |
| E1 | 0.109 | 0.209 | 0.269 | 1 | 0.604 | 1.115 | 0.74 | 1.68 |
| S7 | 0.027 | 0.058 | 0.214 | 1 | 0.644 | 1.027 | 0.916 | 1.152 |
| E6 | -0.02 | 0.044 | 0.21 | 1 | 0.647 | 0.98 | 0.899 | 1.068 |
| A6 | -0.016 | 0.036 | 0.194 | 1 | 0.659 | 0.984 | 0.917 | 1.057 |
| A14 | 0.079 | 0.181 | 0.19 | 1 | 0.663 | 1.082 | 0.759 | 1.544 |
| C1 | 0.049 | 0.116 | 0.181 | 1 | 0.671 | 1.05 | 0.837 | 1.318 |
| A12 | -0.034 | 0.083 | 0.165 | 1 | 0.685 | 0.967 | 0.822 | 1.138 |
| I3 | -0.02 | 0.049 | 0.159 | 1 | 0.69 | 0.981 | 0.891 | 1.08 |
| W2 | 0.069 | 0.177 | 0.151 | 1 | 0.697 | 1.071 | 0.758 | 1.514 |
| I2 | 0.034 | 0.101 | 0.112 | 1 | 0.738 | 1.034 | 0.848 | 1.261 |
| Q2 | -0.01 | 0.03 | 0.107 | 1 | 0.744 | 0.99 | 0.934 | 1.05 |
| N4 | 0.023 | 0.07 | 0.105 | 1 | 0.746 | 1.023 | 0.891 | 1.175 |
| T1 | 0.006 | 0.02 | 0.091 | 1 | 0.763 | 1.006 | 0.968 | 1.045 |
| X1 | -0.017 | 0.06 | 0.083 | 1 | 0.773 | 0.983 | 0.874 | 1.105 |
| W4 | -0.085 | 0.298 | 0.081 | 1 | 0.776 | 0.919 | 0.513 | 1.646 |
| N1 | 0.008 | 0.031 | 0.065 | 1 | 0.798 | 1.008 | 0.948 | 1.072 |
| X9 | 0.016 | 0.068 | 0.054 | 1 | 0.816 | 1.016 | 0.89 | 1.16 |
| M7 | -0.015 | 0.065 | 0.053 | 1 | 0.817 | 0.985 | 0.867 | 1.119 |
| I4 | -0.081 | 0.418 | 0.037 | 1 | 0.847 | 0.922 | 0.406 | 2.093 |
| M8 | 0.03 | 0.183 | 0.027 | 1 | 0.87 | 1.031 | 0.72 | 1.476 |
| H1 | -0.019 | 0.128 | 0.023 | 1 | 0.879 | 0.981 | 0.764 | 1.26 |
| Q4 | -0.014 | 0.101 | 0.02 | 1 | 0.887 | 0.986 | 0.808 | 1.202 |
| T4 | 0.017 | 0.137 | 0.016 | 1 | 0.9 | 1.017 | 0.778 | 1.331 |
| N5 | -0.003 | 0.023 | 0.014 | 1 | 0.905 | 0.997 | 0.954 | 1.043 |
| X3 | -0.004 | 0.037 | 0.012 | 1 | 0.914 | 0.996 | 0.925 | 1.072 |
| K5 | -0.006 | 0.072 | 0.006 | 1 | 0.938 | 0.994 | 0.863 | 1.145 |
| S3 | 0.003 | 0.053 | 0.003 | 1 | 0.959 | 1.003 | 0.904 | 1.112 |
| K6 | -0.02 | 0.399 | 0.003 | 1 | 0.96 | 0.98 | 0.448 | 2.143 |
| G3 | 0.017 | 0.385 | 0.002 | 1 | 0.964 | 1.017 | 0.479 | 2.162 |
| Z2 | 0.001 | 0.052 | 0.001 | 1 | 0.98 | 1.001 | 0.905 | 1.108 |
| Constant | -0.17 | 0.203 | 0.707 | 1 | 0.4 | 0.843 |  |  |

Method = Backward Stepwise (Conditional)
