# Supplementary material for: Correction: Predicting Health Material Accessibility: Development of Machine Learning Algorithms
Source: JMIR Med Inform. 2021 Sep 21;9(9):e33385. doi: 10.2196/33385 (PMC8493462; doi:10.2196/33385)

Multimedia Appendix 3: Originally published Figures 1-4.

Figure 1. Hyperparameter tuning (decision tree)

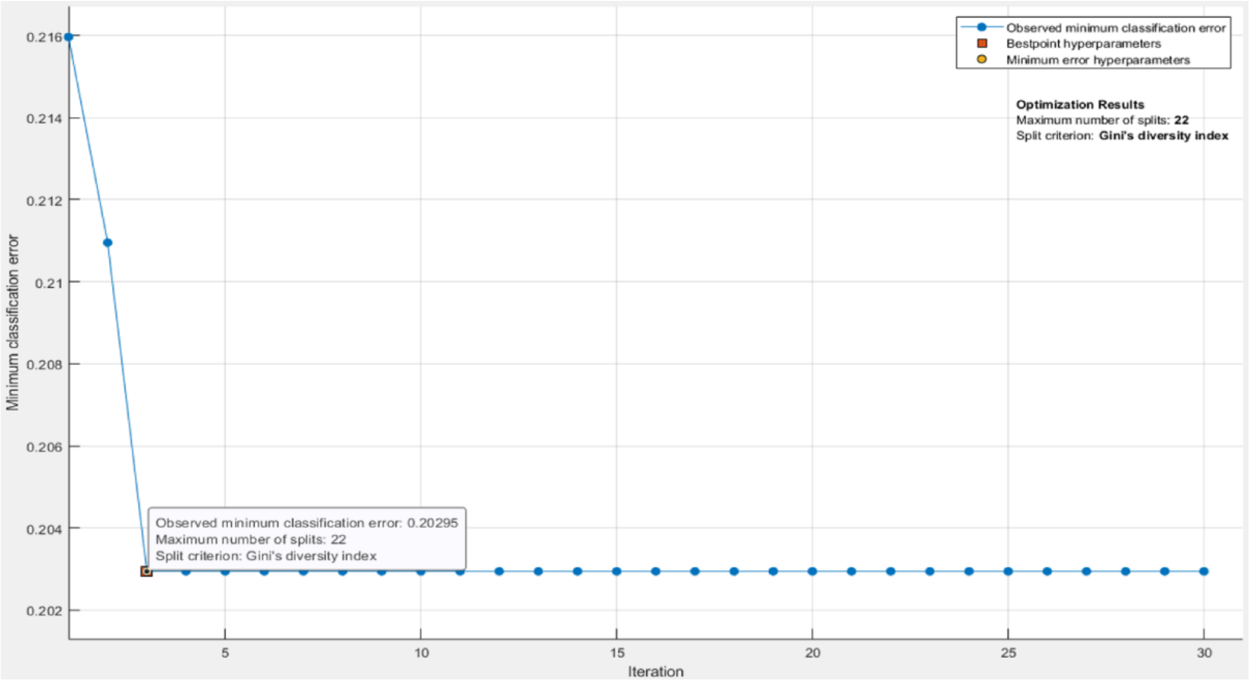

Figure 2. Hyperparameter tuning (ensemble classifier).

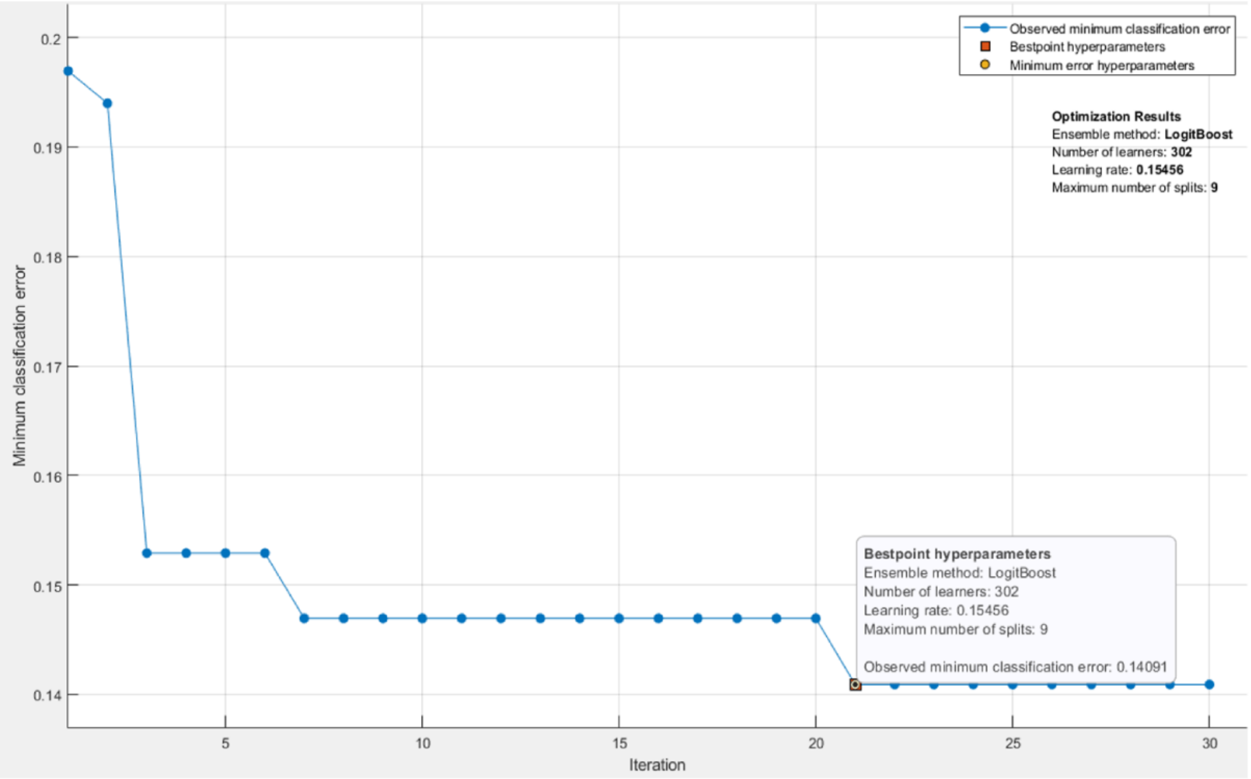

Figure 3. Hyperparameter tuning (support vector machine).

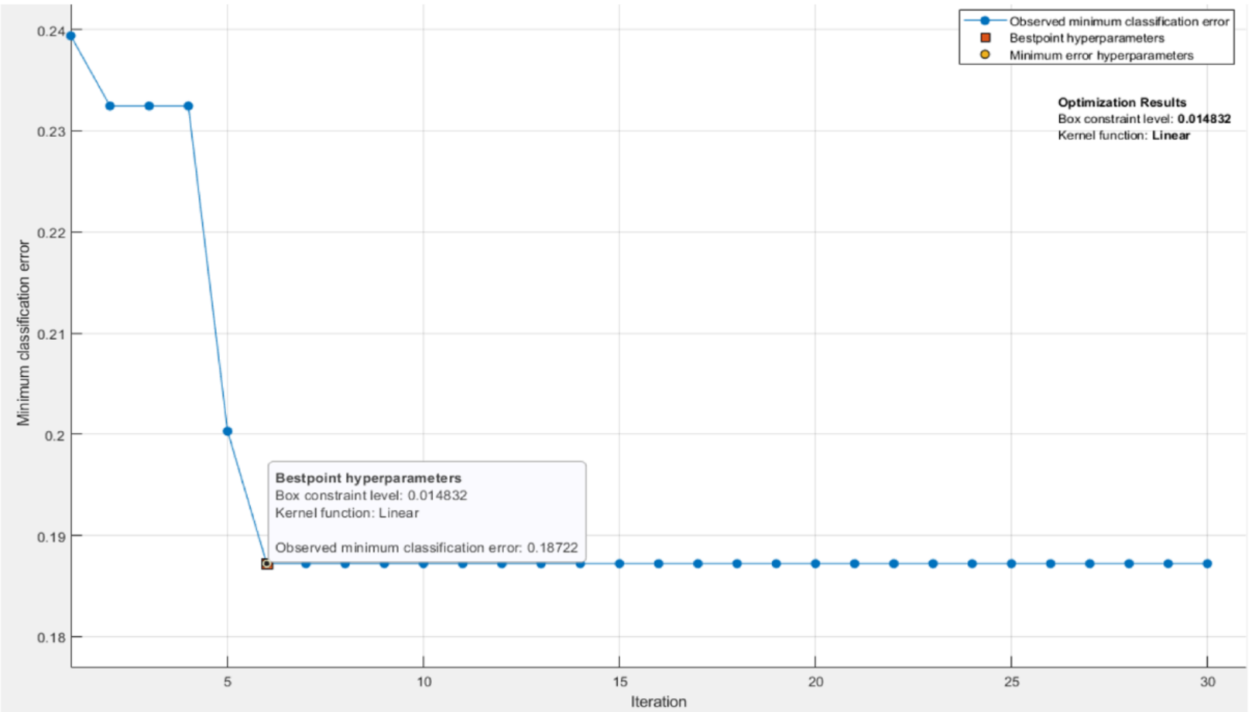

Figure 4. Mean ROC curve for machine learning algorithms. DT: decision tree; LR: logistic regression; ROC: receiver operating characteristic; SVM: support vector machine.

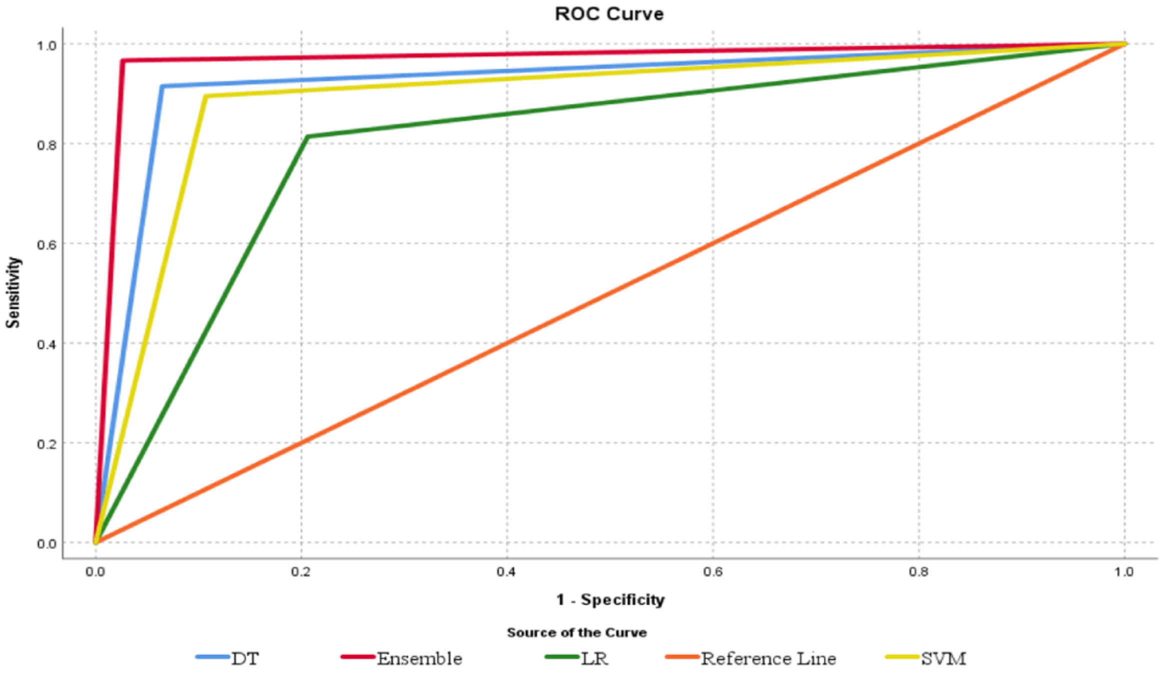

Supplement: Multimedia Appendix 3 [file medinform_v9i9e33385_app3.pdf]
