# Supplementary material for: Correction: Predicting Health Material Accessibility: Development of Machine Learning Algorithms
Source: JMIR Med Inform. 2021 Sep 21;9(9):e33385. doi: 10.2196/33385 (PMC8493462; doi:10.2196/33385)
Supplement: Multimedia Appendix 4 [file medinform_v9i9e33385_app4.pdf]

Multimedia Appendix 4: Originally published Tables 1-5.

Table 1. Performance of the machine learning models using multidimensional semantic features as predictors.

Algorithm	AUC[a], mean (SD)	Sensitivity, mean (SD)	Specificity, mean (SD)	Accuracy, mean (SD)
Ensemble (LogitBoost)	0.97 (0.0121)	0.966 (0.0163)	0.972 (0.0096)	0.969 (0.0114)
Decision tree	0.924 (0.0378)	0.9122 (0.0606)	0.9358 (0.0196)	0.924 (0.0378)
Support vector machine	0.8946 (0.0244)	0.8952 (0.0433)	0.894 (0.0347)	0.8946 (0.0245)
Logistic regression	0.8029 (0.0355)	0.8364 (0.0324)	0.7694 (0.0624)	0.8029 (0.0355)

[a]AUC: area under the operating characteristic curve.

Table 2. Pairwise corrected resampled t test of area under the curve differences (using multidimensional semantic features as predictor variables).

Pairs	Mean difference (SD)	Standard error mean	95% CI	*t* test (*df*)	*P* value
LR[a] vs SVM[b]	–0.0917 (0.0272)	0.0122	–0.1255 to –0.0580	–7.5424 (4)	.002
LR vs DT[c]	–0.1211 (0.0208)	0.0093	–0.1469 to –0.0953	–13.0411 (4)	<.001
LR vs ensemble	–0.1671 (0.0312)	0.0140	–0.2059 to –0.1283	–11.9614 (4)	<.001
SVM vs DT	–0.0294 (0.0385)	0.0172	–0.0772 to 0.0183	–1.7108 (4)	.16
SVM vs ensemble	–0.0754 (0.0200)	0.0090	–0.1003 to –0.0505	–8.4212 (4)	.001
DT vs ensemble	–0.0460 (0.0314)	0.0141	–0.0850 to –0.0070	–3.2711 (4)	.03

[a]LR: logistic regression.
[b]SVM: support vector machine.
[c]DT: decision tree.

Table 3. Pairwise corrected resampled t test of sensitivity differences (using multidimensional semantic features as predictor variables).

Pairs	Mean difference (SD)	Standard error mean	95% CI	*t* test (*df*)	*P* value
LR[a] vs SVM[b]	–0.0588 (0.0308)	0.0138	–0.0970 to –0.0205	–4.2683 (4)	.01
LR vs DT[c]	–0.0758 (0.0595)	0.0266	–0.1497 to –0.0019	–2.8476 (4)	.047
LR vs ensemble	–0.1294 (0.0227)	0.0101	–0.1576 to –0.1012	–12.7580 (4)	<.001
SVM vs DT	–0.0170 (0.0777)	0.0348	–0.1135 to 0.0794	–0.4903 (4)	.65
SVM vs ensemble	–0.0706 (0.0307)	0.0137	–0.1088 to –0.0325	–5.1445 (4)	.007
DT vs ensemble	–0.0536 (0.0553)	0.0248	–0.1223 to 0.0151	–2.1656 (4)	.10

[a]LR: logistic regression.
[b]SVM: support vector machine.
[c]DT: decision tree.

Table 4. Pairwise corrected resampled t test of specificity differences (using multidimensional semantic features as predictor variables).

Pairs	Mean difference (SD)	Standard error mean	95% CI	*t* test (*df*)	*P* value
LR[a] vs SVM[b]	–0.1246 (0.0462)	0.0206	–0.1819 to –0.0673	–6.0367 (4)	.004
LR vs DT[c]	–0.1664 (0.0572)	0.0256	–0.2375 to –0.0954	–6.5073 (4)	.003
LR vs ensemble	–0.2030 (0.0636)	0.0284	–0.2820 to –0.1241	–7.1378 (4)	.002
SVM vs DT	–0.0418 (0.0417)	0.0186	–0.0936 to 0.0099	–2.2438 (4)	.09
SVM vs ensemble	–0.0784 (0.0386)	0.0173	–0.1263 to –0.0305	–4.5440 (4)	.01
DT vs ensemble	–0.0366 (0.0125)	0.0056	–0.0521 to –0.0211	–6.5381 (4)	.003

[a]LR: logistic regression.

[b]SVM: support vector machine.

[c]DT: decision tree.

Table 5. Pairwise corrected resampled t test of accuracy differences (using multidimensional semantic features as predictor variables).

Pairs	Mean difference (SD)	Standard error mean	95% CI	*t* test (*df*)	*P* value
LR[a] vs SVM[b]	–0.0917 (0.0272)	0.0122	–0.1255 to –0.0579	–7.5357 (4)	.002
LR vs DT[c]	–0.1211 (0.0208)	0.0093	–0.1469 to –0.0953	–13.0459 (4)	<.001
LR vs ensemble	–0.1663 (0.0317)	0.0142	–0.2056 to –0.1270	–11.7369 (4)	<.001
SVM vs DT	0.0294 (0.0385)	0.0172	–0.0772 to 0.0183	–1.7108 (4)	.16
SVM vs ensemble	–0.0746 (0.0190)	0.0085	–0.0982 to –0.0510	–8.7800 (4)	.001
DT vs ensemble	–0.0452 (0.0328)	0.0146	–0.0859 to –0.0045	–3.0860 (4)	.04

[a]LR: logistic regression.

[b]SVM: support vector machine.

[c]DT: decision tree.
